# An automated framework for QSAR model building

**DOI:** 10.1186/s13321-017-0256-5

**Published:** 2018-01-16

**Authors:** Samina Kausar, Andre O. Falcao

**Affiliations:** 1 LaSIGE, Departamento de Informática, Faculdade de Ciências, Universidade de Lisboa, 1749-016 Lisbon, Portugal; 2 BioISI: Biosystems and Integrative Sciences Institute, Faculdade de Ciências, Universidade de Lisboa, 1749-016 Lisbon, Portugal

**Keywords:** Quantitative structure–activity relationship (QSAR), Machine learning, Feature selection, Variable importance, Random forests, Support vector machines, KNIME, Data set modelability

## Abstract

**Background:**

In-silico quantitative structure–activity relationship (QSAR) models based tools are widely used to screen huge databases of compounds in order to determine the biological properties of chemical molecules based on their chemical structure. With the passage of time, the exponentially growing amount of synthesized and known chemicals data demands computationally efficient automated QSAR modeling tools, available to researchers that may lack extensive knowledge of machine learning modeling. Thus, a fully automated and advanced modeling platform can be an important addition to the QSAR community.

**Results:**

In the presented workflow the process from data preparation to model building and validation has been completely automated. The most critical modeling tasks (data curation, data set characteristics evaluation, variable selection and validation) that largely influence the performance of QSAR models were focused. It is also included the ability to quickly evaluate the feasibility of a given data set to be modeled. The developed framework is tested on data sets of thirty different problems. The best-optimized feature selection methodology in the developed workflow is able to remove 62–99% of all redundant data. On average, about 19% of the prediction error was reduced by using feature selection producing an increase of 49% in the percentage of variance explained (PVE) compared to models without feature selection. Selecting only the models with a modelability score above 0.6, average PVE scores were 0.71. A strong correlation was verified between the modelability scores and the PVE of the models produced with variable selection.

**Conclusions:**

We developed an extendable and highly customizable fully automated QSAR modeling framework. This designed workflow does not require any advanced parameterization nor depends on users decisions or expertise in machine learning/programming. With just a given target or problem, the workflow follows an unbiased standard protocol to develop reliable QSAR models by directly accessing online manually curated databases or by using private data sets. The other distinctive features of the workflow include prior estimation of data modelability to avoid time-consuming modeling trials for non modelable data sets, an efficient variable selection procedure and the facility of output availability at each modeling task for the diverse application and reproduction of historical predictions. The results reached on a selection of thirty QSAR problems suggest that the approach is capable of building reliable models even for challenging problems.

**Electronic supplementary material:**

The online version of this article (10.1186/s13321-017-0256-5) contains supplementary material, which is available to authorized users.

## Introduction

### Background

The advantages of automation of repetitive tasks in the laborious drug discovery process are numerous and include increased research quality by reducing error along with significant time saving, boosted up productivity, and capacity to name a few. In this era where large amounts of data are produced every day and large computational resources are available, the introduction of machine learning approaches has significantly automated the drug discovery procedure and provides a faster alternative for ultrahigh-throughput screening of large databases of chemical molecules against a biological target [1–3].

Machine learning approaches are being applied in the drug discovery cycle to produce a robust model, capable of empirical predictions of biological properties of candidate compounds for new therapeutic molecules. Many successful studies have been reported in the literature which attests the importance of machine learning approaches combined with traditional practices to approach medicinal chemistry challenges [4]. In traditional lab work methodologies, many expensive tests are often required which many times include animal testing to provide information about human safety for suggested chemicals. The legislation does not support such frequent experiments on laboratory animals, but rather promotes the sharing of data to the use of integrated alternative in-vitro and in-silico strategies of toxicokinetics [5–7]. Currently the Avicenna Research and Technological Roadmap, funded by the European Commission, strongly suggests the use of in-silico techniques coupled with clinical trials [8]. This framework describes strategic priorities to establish the safety assessment of new medical interventions and at the same time minimizes the ethically concerned activities such as the animal or human experimentation.

Several available in-silico QSAR models based tools are widely used to screen very large databases of compounds in order to determine toxicity or any desired biological effects of chemical molecules based on their chemical structure [9, 10]. The well-characterized internationally accepted validation principles for creating validated models have been used by regulatory agencies of United Sates (US) and gaining a boost in the European Union (EU) too [8, 11–13]. In the EU, the standard recommendations of chemicals risk assessment by regulatory QSAR models has been set by the Registration, Evaluation, Authorization, and Restriction of Chemicals (REACH) [14] and the Organization for Economic Co-operation and Development (OECD) [15]. The progress of such projects highlights the increased importance of productivity gains from fully accessible automation in the drug discovery and QSAR modeling fields.

These days, the aim of pharmaceutical projects is the integration of complex non-homogeneous data to build global models intended to be applicable within wide ranges of chemical space. However, with the passage of time, there is an exponentially growing amount of synthesized and known chemical compounds data being added to the many existing molecule databases, public or private. This rise of available data is producing new opportunities to build models with broader applicability domains while at the same time challenging the existing models, as wider data sets allow for a more extensive testing and validation of previous in-silico screening efforts. From these databases, data can easily be explored to build QSAR models based on available structural properties of the compounds that correlate with their biological activity [16–18]. These models can also be used as an efficient tool to improve the understanding of biological processes. Also, well-trained and properly validated models are reliable for automated prediction of physiological characteristics of new compounds to assist the experimental drug discovery process by decreasing the time of the initial screening stages [19–22].

The QSAR/QSPR modeling “life cycle” involves some standard steps, critical for reliable model building. These steps include (1) model building by the application of one or several machine learning approaches, (2) model validation with an internal test set to assess its quality (3) model selection according to the results of the internal validation procedure, and (4) model validation with an external test set (Independent Validation Set) to ascertain its predictability of the properties of compounds never tested in model building and thus giving a more reliable measure of the selected model quality [4, 22]. It is also important to consider model updating as new data may become available. This repetitive nature of QSAR/QSPR modeling “life cycle” highlights a fundamental requirement of automation of critical steps with well-defined input, outputs, and success criteria in both the drug discovery industry and biomedical research. To achieve this objective, it is fundamental to have a scrutinizable procedure for applying to a variety of problems. Automating such procedures in the form of a reusable workflow is a reachable goal with current technology, provided that a reliable method is extant and applicable to a wide range of problems. Such automation would reduce the necessary and often tedious labor of model building, while at the same time guaranteeing that, for the available data, a quality model is reached.

Over the past decade, attempts have been made to attract the attentions towards the need of automation of the QSAR modelling process. More recently, Dixon et al. [23] developed a machine-learning application (AutoQSAR) for automated QSAR modeling. It is unable to access data directly from online repositories and users required deep understanding to prepare a curated and standardized data set before modeling by AutoQSAR. eTOXlab [24] which is another framework allows automated QSAR mainly by a command line interface. Python programming skills are necessary to work with eTOXlab. An interesting alternative of integrated solution for fully automated modeling is OCHEM [25] but it’s online nature makes it unsuitable for using it with private/sensitive data sets, which demand better privacy and safety independent of third party. Cox et al. [26] designed a Pipeline Pilot web application (QSAR Workbench). This application makes the built models available to all users in Pipeline Pilot [27], which is not freely available to the vast scientific community. The Automated Predictive Modeling, another modeling system [28], demands expert technical skills and significant resources for model development and maintenance.

### Objectives

Some of the major pinpointed gaps in the above discussed software packages include lack of fully automated process, require that users have a thorough understanding of the data and modeling problems and several require computer programming and/or machine-learning knowledge, complex parameterization to customize complex modeling algorithms, and most do not give full access to view the intermediate results at each step of the modeling. Also to the best of our knowledge, none of these packages provide a facility to check overall data quality/feasibility to produce a robust QSAR model (data modelability), which can be an important measure to minimize time and computational cost. In the current work, we developed an open source automated QSAR modeling system that addresses these issues by providing better solutions for expert and non-expert users. The key ideas behind structuring the presented automated QSAR modeling workflow platform are:

- It should be freely available and support any operating system with easy installation.
- Should be easily be applied for fully automated QSAR modeling by directly accessing up to date data from online molecules databases or by using private data sets.
- Provide automated data curation facility including removal of irrelevant data by selecting only the bioactivity type of interest, filtering out missing data, handling of duplicates (e.g. same or two experimental records: same structure) and dealing with several forms of the same molecule (including salt groups).
- Reliably perform most critical tasks of QSAR modeling including descriptor/fingerprints calculation, feature selection, model building, validation, and prediction.
- Make a prior estimation of the feasibility of any given data set to produce a predictive QSAR model before the time-consuming process of feature selection, model building and validation.
- It should adopt the best optimized feature selection methodology to select the adequate features for each problem. This is a critical task necessary to avoid over-fitting and to have a better understanding of the data, the model and the factors involved.
- The application must follow the same protocol of training series to re-train and update models with new molecules as they become available and to make external predictions.
- For different applications and reproduction of historical predictions, all outputs of intermediate tasks and each previous version of models must be stored on local machines.
- Regarding extensibility, the framework should provide useful starting points for performing customization to modify and further extend the existing workflow by domain specific interests.

Many research labs aim to develop their own complete workflow by using workflow automation tools for a broader domain of related biological problems [29–32]. Some of the more popular workflow frameworks include Taverna [33], Pipeline Pilot [27], Galaxy [34], Kepler [35], Loni Pipeline [36] or the KoNstanz Information MinEr (KNIME) [37]. These well-deployed workflows with graphical user interface provide a clear view of the running process rather than working as a black box, or with complex and opaque code. Moreover, it is an efficient way to manage complex chemical data to help standardize procedures, automate laborious procedures, and assist in data analysis [29]. For the current study, we have selected KNIME, an open source data-mining framework developed by the Nycomed Chair for Bioinformatics and Information Mining at the University of Konstanz to manipulate and analyze data with a strong emphasis on chemical manipulation and information management. KNIME has made it easy to perform the calculation of molecular descriptors to quantify molecular structures, evaluation of chemical similarity and other cheminformatics problems [CDK [38], RDKit [39], Schrodinger [40, 41], ChEMBL [42], OpenPHACTS [43], BioSolveIT (http://www.biosolveit.de/KNIME)].

The developed open source automated QSAR modeling KNIME workflow embeds all tools necessary to perform all steps of the QSAR life cycle by following best practicing methods [22, 44]. This designed workflow can easily be applied to build the predictive QSAR models reliably by directly accessing online manually curated databases or using users own private data without having expertise in machine learning/programming. In this work, we illustrate and describe a model building workflow with an optimized feature selection methodology and show its application in real world examples, by directly fetching binding data for thirty different QSAR problems from an online manually curated database (ChEMBL [42]) and building models using runtime prepared processed data. The workflow, given a target or problem, automatically accesses and processes molecular data, calculates descriptors and fingerprints, evaluates data set modelability, selects optimized set of features by using an established methodology [45] and follows an unbiased standard protocol [22, 44] of QSAR model building by external and internal validation. The objective of this work is not to highlight the predictive power of the presented models but rather to elaborate a reliable methodology to automate the production of models with good predictive qualities for very difficult problems. Nonetheless, the quality of the results suggests that the approach is capable of building reliable models for a large variety of problems.

## Automated model building

**Fig. 1:**
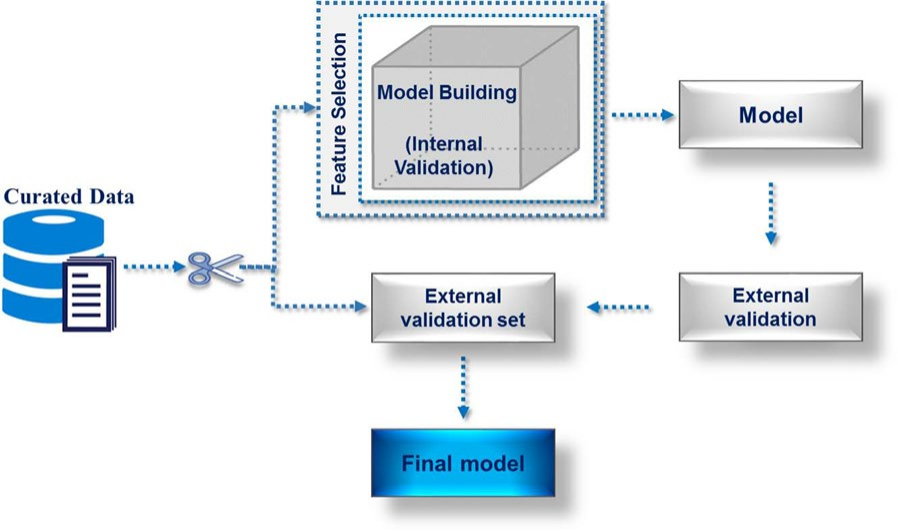
Overview of automated QSAR modeling workflow

The main focus of the current work is to present an implementation of a well-defined and efficient modeling procedure capable of building robust and reliable models and validate them both internally and externally. To accomplish this it was necessary to address two critical issues in QSAR modeling. The first one is to know how to deal with high dimensional data by identifying and selecting the subset of descriptors sufficient to predict the desired biochemical property. The second aspect in a modeling workflow is model validation, so that the model results can be unbiasedly assessed. This will ultimately qualify the applicability of the model for activity prediction of external compounds in drug discovery processes [22]. An overview of the standard protocol of automated QSAR modeling workflow is shown in Fig. 1. This workflow starts with data preparation and data quality validation, data curation that includes gathering molecular structures and corresponding biological activity data for a specified target. Furthermore, to quantify various features of molecular structures a variety of chemical descriptors are computed. Before proceeding to the time-consuming trials of feature selection, model building and validation, data modelability evaluation is performed. Difficult data sets will not be recommended to model. After this step, the feature selection process follows, so as to identify an optimized non-redundant set of variables that can lead to best models. This critical step not only provides a better understanding of generated data but also improves the prediction performance of relevant predictors [45]. This latter phase typically involves extensive testing of different models with an increasing set of variables. Finally, when a relevant and reduced set of variables has been determined, it can be used to develop the final QSAR model by following a rigorous internal and external validation process without compromising model quality assessment.

### Architecture

**Fig. 2:**
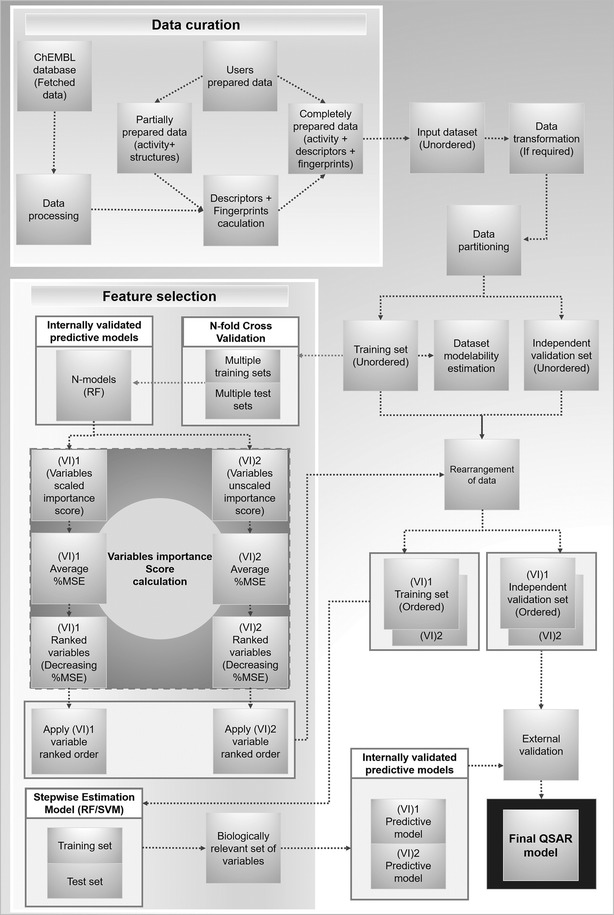
Automated QSAR modeling methodology

This QSAR modeling workflow uses several customized nodes of (KNIME version 3.2) and is able to access online databases with millions of bioactive compounds. KNIME nodes can perform an extensive set of functions for many different tasks such as read/write data files, data processing, statistical analysis, data mining, and graphical visualization. Moreover, to reduce the complexity of large complicated workflow, a particular part of the workflow (sub-workflows) can be isolated in meta-nodes. The developed workflow aims at the simplification and automation of the QSAR model building. An overview of the implemented methodology is shown in Fig. 2 (see full KNIME view in Additional file 1: Figure S1). The complete process is divided into several systematic tasks of QSAR modeling including (a) data access and processing, (b) descriptors calculation, (c) data set modelability estimation (d) feature selection, (e) model building and (f) validation, along with adequate data visualization. Each of these subtasks is enclosed within the KNIME meta-nodes that are isolated from the rest of workflow enabling easy parameterization with a user-friendly configuration interface. The details of each task are covered in the following sections.

### Data access and processing

**Fig. 3:**
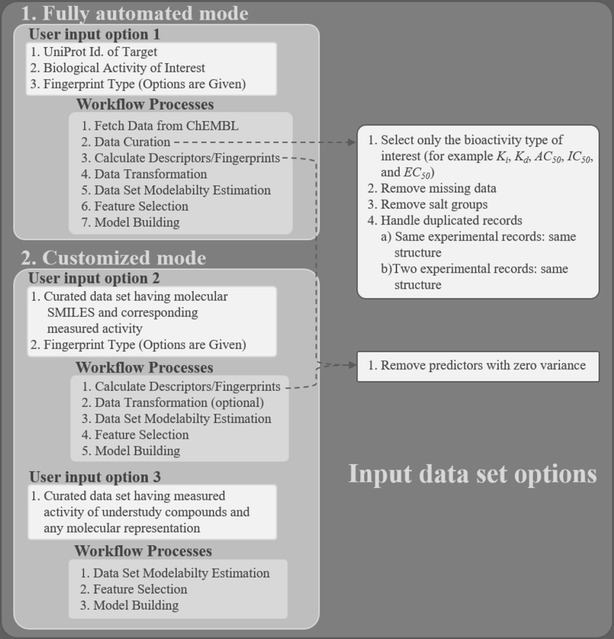
Input data set options. Overview of possible ways to submit input data to the automated QSAR modeling workflow

There are typically two different alternatives for data set construction in model building, either the user has its own private data set with measurements curated from different sources or measured in the lab, or else retrieves the information from an available online data repository, that is continuously being updated by dedicated teams. The proposed workflow is able to encompass both approaches, giving the user the ability to use its own data set (with optional structural and descriptor calculation) or use an online repository (Fig. 3).

Nowadays, there are several large open source databases with annotated bioactivities for small molecules, with comprehensive information on biological properties of millions of chemicals. This wide data availability is one of driving forces beneath this effort. Most popular molecular databases like PubChem [46, 47], PDSP Ki [48], and ChEMBL [42] have become leading cheminformatics resources. The “Fully Automated” mode focus on ChEMBLdb by taking advantage of KNIME facility to access ChEMBL data. KNIME provides two built in nodes “ChEMBLdb Connector” and “ChEMBLdb Connector Input” to interact with RESTful and XML web services of ChEMBLdb. This facility for other chemical databases is not available yet. However, the ChEMBL database of more than 1.5 million bioactive compounds and 9000 biological targets is capable to provide an ample variety of problems. In KNIME, the “ChEMBL database” meta-node encapsulates a complete workflow to access data from ChEMBLdb, data processing, and descriptor and fingerprint calculation (Additional file 1: Figure S2). Hence, users can quickly access ChEMBLdb chemicals data for any target of interest by just a simple query of the desired UniProt ID and associated biological activity. The data obtained from ChEMBL may contain information related to all available biological activities extant for a given biological target (for example $K_{i}$, ${K_{d}}$, ${AC_{\textit{50}}}$, ${IC_{\textit{50}}}$, and ${EC_{\textit{50}}}$). This retrieved data is processed by retaining only the user’s requested biological activity type records, and other relevant information related to chemical structures and assays. As the objective is to quantify a ligand–target interaction (activation or inhibition of the target), therefore any activity value can be utilized to count data related to the hypothesis. Overall data curation also includes the identification of missing data and duplicates (current year records are considered in two experimental records for same molecular structure) and dealing with several forms of the same molecule (including salt groups).

### Descriptors calculation

The usage of descriptors and other computational representations of molecular structures is one of the principal methods applied to screen the new active molecules. The current workflow automatically calculates several molecular descriptors and structural characteristics for the retrieved molecules.

Along with this facility of online data access, users can also submit their fully prepared data file by using other input data set options with any types of descriptors calculated elsewhere. The workflow is able to use RDKit for descriptor calculations and can compute as well nine different fingerprint types, including Morgan, FeatMorgan, AtomPair, Torsion, RDKit, Avalon, Layered, MACCS and Pattern [39].

### Data transformation and data partitioning

Scaling/transformation of the response variables (associated bioactivities) can be performed to standardize highly varying values in raw data for proper training of predictive model, where often data is transformed with a logarithmic function. This transformation can be skipped if data is already normalized. For the assessment of the applicability (prediction error) of the developed QSAR model, at this stage, the submitted data (either by automated retrieval from an online source, or by direct loading from a private data set), is divided into training set and Independent Validation Set (IVS) through a random partition. The training set is further used in N-fold cross validation process for internal model evaluation and selection while the IVS data is used to perform an unbiased model validation after the best model is built and selected. The latter is never used for any feature selection or model training procedure. So as not to bias the results.

### Data set modelability estimation

Predictive performance of QSAR models highly depends upon different characteristics (e.g., size, chemical diversity, activity distribution or presence of activity cliffs) of various data sets [49–51]. It may not be always possible to build reliable QSAR models for certain data sets. To identify difficult problems, recent studies have introduced the concept of “data set modelability” meaning a prior estimate of the feasibility to obtain robust QSAR models by using a given descriptor space for data set of molecules [52–54]. The key idea behind this concept is based on the similarity principle that states that ‘similar compounds typically exhibit similar activity’ [55]. However, For every compound in a given data set, the nearest neighbors, i.e., compounds with the smallest distance from a given compound should possess similar activity. If the target property values for highly similar compounds are significantly different, then it means that the problem is probably hard to solve and most approaches will not be able to model it.

In the presented workflow, we followed a well established k-nearest neighbors approach based criteria, the modelability index (MODI) [53]. Golbraikh et al. [53] proposed several statistical criteria for estimating the feasibility of classification [e.g., data set diversity (*MODI_DIV*), activity cliff indices (*MODI_ACI*), correct classification rate (*MODI_CCR*)] and regression [similarity search coefficient of determination ($MODI\_q^{2}$ and $MODI\_ssR^{2}$)]. MODI is calculated as the Leave-One-Out (LOO) cross validation coefficient of determination of a simple k-Nearest Neighbours approach for data classification or regression over the training set, where k is typically either 3 or 5. MODI is fast to compute and helps modelers to quickly evaluate whether any given chemical compound data set can be modelled, giving an estimation of the predictability of the computed models before the actual modeling takes place. Data sets with very low MODI index are not recommended for model building, as a low MODI index informs the user that additional data processing and manual curation may be required. However, according to the suggested MODI score for regression problems [53], in the automated QSAR modeling workflow (Additional file 1: Figure S1), we suggest a MODI score to be > 0.45 for reaching a model with acceptable predictability (PVE $\ge 0.60$).

### Feature selection

The goal of QSPR/QSAR models is to correlate the molecular structures with their physiochemical/biological properties [20–22]. There are three main difficulties to achieve this task: (1) how to quantify molecular structure; (2) identify which are the relevant structural descriptors (or structure derived) that are the most adequate for the problem at hand; and (3) how to actually map the descriptors selected to the property being modeled [20–22, 56, 57]. Molecular descriptors can approximate most structural properties and a huge corpus of literature is extant on this subject [58]. Currently the number of chemical descriptors is so large that one of the biggest problems is selecting the most adequate features for each problem [58, 59]. Several issues typically need to be addressed in feature selection when the number of available variables is very large [60–62]. Some of the typical problems are:

- Some descriptors appear highly correlated.
- In several biological contexts no hypothesis is available about target structure for inferring binding activity.
- Having many descriptors many times just do not improve the model quality, as the number of features advances, the number of spurious correlations increases as well and adding redundant or irrelevant variables to the model do not increase the model predictive abilities.
- Sometimes the given descriptors are not, by themselves, able to contribute to modeling activity, but by combining them with other available descriptors, may sometimes increase the model prediction capabilities.
- The identification of a limited set of descriptors from the available list is many times necessary to avoid over-fitting, allow the desired physicochemical property to be adequately predicted by the constructed model and to have a better understanding of the models and the factors involved.

For the purpose of feature selection, several statistical and non-linear machine learning methods have been employed in QSPR/QSAR modeling as filter techniques. Some direct feature filtering approaches includes correlation matrix, Fisher’s weight, Principal Components Analysis or Weighted Principal Components Analysis or Partial Least Squares (PCA/WPCA/PLS) loadings, regression coefficients, variable importance in PLS projections [VIP]) and Random Forest (RF). Some other are iterative methods for example, Ordered Predictor Selection-Partial Least Squares (OPS-PLS), Sequential Forward/Backward Selection, randomized methods that combine PLS with Genetic Algorithms (GA) or Monte-Carlo algorithms [45, 63–66]. The direct filter methods are simpler and faster selecting variables, since they require only a metric calculation (a coefficient or weight) and the application of a cut-off value to determine the rejection of some variables due to the low importance to the model construction. Iterative methods have high computational cost, since most of them use filter methods in iterative ways or in combination with machine learning techniques. However, to deal with high dimensional data, the best-optimized methodology is always required to select the minimum subset of descriptors to predict a certain property with a good performance, less computational/time cost and in a more robust way. The application of non-linear machine learning algorithms to explore the non-linear relationships between descriptors and biological activities is increasing within the QSAR community [67, 68]. For feature selection in predictive models, we implemented a RFs voting procedure that can be used for the variable rankings according to their importance in RFs models [45, 65, 69]. In this ensemble method, each variables importance score is calculated by several available variable importance’s (VI) measures . One of the widely used VI measure in the regression problems is increase in mean of the error of a tree “Mean Squared Error (MSE)”, which explains how much prediction error increases with the random permutation of given variable while keeping all others unchanged in a node of a tree [65, 69–71]. Moreover, RF provides two options to fetch the VI score, which includes scaled and unscaled importance score. The scaled importance (also called z-score) is the default output of the randomForest function, which is obtained by division of the raw/unscaled importance by its standard error.

1 $$Z_j = \frac{(VI(X_j))}{\frac{\widehat{\sigma }}{\sqrt{ntree{}}}}$$

However, some studies indicate that the unscaled importance VI (Xj) has better statistical properties and recommended for regression problems [72, 72, 73].

2 $$VI(X_j ) = \frac{\sum _{t=1}^{ntree} VI^{(t)}(X_j )}{ntree}$$

The current workflow followed the best performing RF based feature selection method, which is a hybrid approach [45]. The principle of this hybrid technique is to get: (1) possible set of variables, most relevant to the property of interest by using the variable importance (VI) function of RFs and (2) obtain the minimal set of features with a possibly best predictive performance along with unfavorable ratio between the number of predictors and number of observations. Practically, this approach counts variable importance by calculating the average mean squared error (MSE) provided by RF from a series of runs as a tool to rank the predictors. Hence, the VI based ranked variables can be feed to any machine learning algorithm to build the stepwise predictive models to find a better balance between the biologically relevant set of features and prediction error (RMSE).

### Model building

#### Model without feature selection

To verify performance of the applied feature selection method, it is necessary to assess model predictive behavior without any feature selection. Hence, developed QSAR modeling workflow, build a model with whole set of descriptors to confirm that elimination of irrelevant or non informative variables is improving predictive power of given model.

#### Model with feature selection

Automated QSAR modeling workflow follows a RF based feature selection method and provide ranked order of variables without eliminating any variable. These ranked variables are sequentially added to the learning algorithm to find the most relevant set of predictors leading to the model of smallest error rates.

The most employed machine learning approaches used in in-silico drug design are artificial neural networks (ANN), support vector machines (SVM), decision trees (DT), random forests (RF) and k-nearest neighbors (KNN) [4, 63]. Among the mentioned methods, the use of SVM to build QSAR models has become very popular in the last years [74–77]. Moreover, many studies also explain the suitability of RF for high dimensional QSAR/QSPR datasets [45, 70, 78]. Hence, SVM [79] and RF [70], non-linear supervised learning methods are made available in the QSAR modeling workflow. This is mainly due to the fact that these methods are robust in finding good modeling approaches in complex situations where the number of variables is very large and the number of instances is typically small. In such situations, many other machine learning methods (decision trees, neural nets, or linear models) can easily over fit, producing models unable to generalize outside the training space. Nonetheless, other algorithms can easily be used within KNIME, either through its customized nodes or by linking KNIME to R modules where most modeling approaches have been implemented.

To evaluate models predictability, data is split into training and test set to generate and validate stepwise estimation model by sequentially feeding ranked variables. The best features based internally validated model is finally presented for external validation.

### External validation and model applicability domain

It is crucial to define the applicability domains of developed models by a critical step of external validation by using an IVS, which is not used in any part of the training process. In the developed workflow, a stringent protocol [22] of model validation is followed to ensure robustness and predictive power of the constructed models. The evaluation of the models’ fitness is performed by comparing the proportion of the variance explained (PVE) by the predictive model, and the root mean squared error (RMSE) [80] (see Eqs. 1 and 2). Externally evaluated final models can be used as a tool for external prediction and virtual screening.

3 $$PVE= 1 - {{\frac{{\sum _{{i = 1}}^n {{{\left( {{y_i} - {{\hat{y}}_i}} \right) }^2}} }}{{\sum _{{i = 1}}^n {{{\left( {{y_i} - {{\bar{y}}_i}} \right) }^2}}}}}}$$

4 $$RMSE= {\sqrt{\frac{1}{N}{\sum \limits _{i = 1}^N {(y_{i} - \hat{y}_{i} } })^{2} } }$$

In Eqs. 3 and 4, $y_i$ and ${\hat{y}}_{i}$ are the measured and predicted biologically associated values for compound *i*, respectively, and $\bar{y}$ is the mean of all activities from the compounds in the data set.

Nevertheless, in external predictions, the new data has molecules not present in the training set, therefore some predictions made with the model can be unreliable. This issue may be addressed by training models with a larger size and increased diversity, which many times is not an option in QSAR studies, or to circumscribe the model by defining its applicability domain (AD) in the chemical space [81, 82]. In the model AD, a similarity threshold between the training and validation set is established to flag the newly encountered compounds for which predictions may be unreliable. If the similarity between the training and validation set or new chemical is beyond the defined similarity threshold, the new compound is accounted to be outside the AD and the prediction is considered unreliable [81, 82]. In this QSAR modeling workflow, a well-established method [82] is used to define the domain of applicability of the built models based on the Euclidean distances among the training data and IVS.

### Extensibility

The main modeling workflow is subdivided into several tasks. Each subtask is performed by small workflows that are developed and encapsulated within meta-nodes to establish independent processing and analysis (Additional file 1: Figure S1). The subdivision of the complete modeling process in QSAR modeling workflow architecture provides several advantages including (a) it reduces the complexity of modeling framework (b) improves the understanding of the implemented machine learning procedure and (c) increases the flexibility for future modification of the workflow. Hence, users can easily modify and further extend the presented workflow by domain-specific interests to add new features.

## Results

### Workflow implementation

Each task during drug designing from data preparation to model development and validation is critical to the accuracy of the predictive power of QSAR models [22]. The first stage of data preparation includes data collection, data cleaning by removing unwanted data, and appropriate molecular representation of underlying chemical compounds. In the second step the curated data is evaluated by data modelability criteria to check either given data set is reasonable to generate a QSAR model with significant predictive power. The third step includes extraction of more relevant biological features entitles as feature selection. Finally, model development and validations emphasize on a standardized process of internal and external model validation. QSAR modeling workflow is developed especially focusing on these mentioned major tasks to develop best-established methodology based framework.

#### Input data parameters

**Fig. 4:**
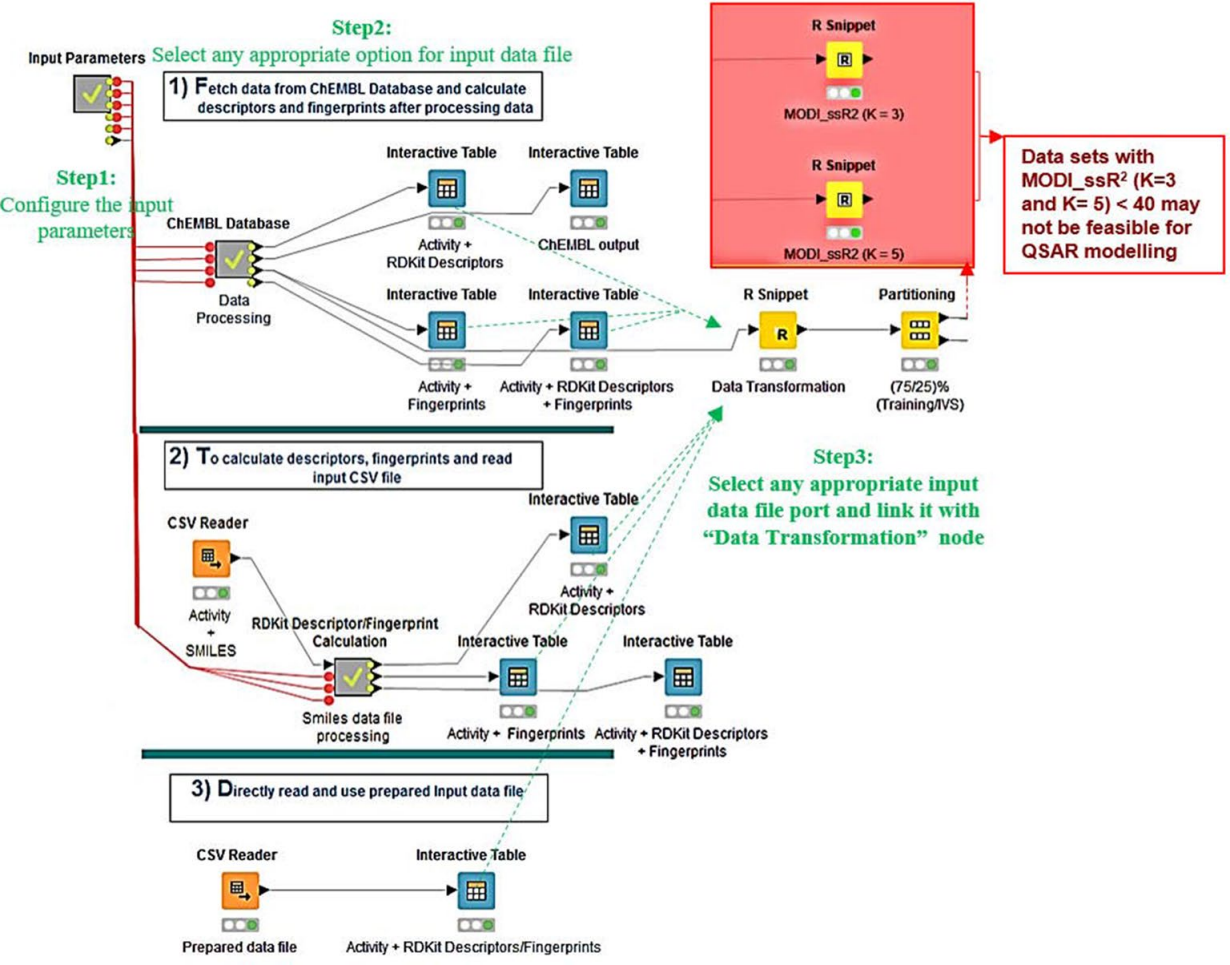
Input parameters. Input configurations required before to run the workflow

To run automated QSAR modeling workflow, simple settings of “Input Parameter” meta-node (Fig. 4), like the choice of the target protein (name and UniProt ID), molecular fingerprints, nfold value, working directory path and the type of activity measures are required to build the best possible predictive model in very short time. No parameter is required to get RDKit descriptors for the given target; these are calculated by using the RDKit nodes embedded inside “ChEMBL Database” meta-node (Additional file 1: Figure S2). Optional parameters node “Machine learning algo” provide the choice of machine learning algorithm (by default = SVM) (Additional file 1: Figure S1).

#### Input data set options

Automated QSAR modeling workflow provides three options to take input data files (Fig. 3). The first option provides a “Fully Automated” mode, which directly accesses data from ChEMBL database with a simple query of UniProt accession number of a target protein and associated bioactivity type. No deep understanding of data is required for the first option.

There are two other alternatives for modelling within “Customized” mode, if the user wants to work with personal data sets, and none of them requires ChEMBL data retrieval. Within “Customized” mode, the two alternatives deal with different available structural and descriptors-based information within the data sets, as the user is able to provide most of the data. Users with preliminary knowledge of their understudy problems can choose option 1 of “Customized” mode to process the known list of curated molecules. In the case of a thorough understanding of given modeling problem, where the user has previously computed the necessary molecular representation (with chemical descriptors or other structural information) the “Customized” mode option 2 bypasses all the descriptor computation phases and proceeds directly to model building. Hence, by adding flexibility in the way the user is able to provide input data, this constructed framework is able to cover some of the most common needs of modelers.

#### Data set retrieval and data pre-processing

In the “Fully Automated” mode to fetch data from ChEMBL the “ChEMBL Database” meta-node is developed in a given workflow (Fig. 4 and Additional file 1: Figure S2). This meta-node can automatically prepare standard input data sets to explore a ChEMBLdb reported compounds–chosen receptor interaction by quantification of bioactivity of molecules.

In ChEMBLdb, different measures for binding affinities have been standardized, some of them remain more used like the half-maximal effective concentration (${EC_{\textit{50}}}$), the half-maximal inhibitory concentration (${IC_{\textit{50}}}$) and the inhibitory constant ($K_{i}$). ${EC_{\textit{50}}}$ value represents the molar concentration (M = mol/L) of an agonist that produces half of the maximal possible effect of that agonist. The simple definition of ${IC_{\textit{50}}}$ is a molar concentration of an antagonist that reduces the response to an agonist by 50%. Moreover, it can be explained as the molar concentration of an unlabeled agonist or antagonist that inhibits the binding of a radio-ligand by 50%; or can be considered as the molar concentration of an inhibitory agonist that reduces a response by 50% of the maximal attainable inhibition [83, 84]. $K_{i}$ value is used to quantify a ligand-receptor interaction based on the equilibrium dissociation constant (K). Hence, smaller the $K_{i}$ value is associated with higher ligand-receptor binding affinities [68, 85].

In this machine leaning pipeline, the focus is to set a standard protocol of regression problem based on any measure to predict the tendency of chemical molecules to either activate ($K_{i}$, ${K_{d}}$, ${AC_{\textit{50}}}$, or ${EC_{\textit{50}}}$) or inhibit (e.g., those with ${IC_{\textit{50}}}$ values/$K_{i}$ values) a selected target. The “ChEMBL Database” meta-node returns ChEMBL retrieved data (ChEMBL ID., reference, bioactivity type, assay description, activity value, and smiles strings), the calculated descriptors, and fingerprints data sets. Both the data sets of descriptors and the fingerprints can be used for further processing and modeling.

#### From data to validated models

Data pre-processing occupies a large time cost in QSAR modeling process. Many nodes are available in KNIME for data manipulation including row/column filtration, merging, splitting, concatenation and joining, type conversion and data transformation, row groping and aggregation, and data table pivoting. Moreover, to process and handle large amount of data on a standard computer, KNIME also provides efficient memory management architecture. Hence, developed automated QSAR modeling workflow incorporates these all advantages of data processing and handling. It automatically fetches and processes data in an efficient way with the combinations of KNIME built in nodes with in this workflow (Additional file 1: Figure S2). Data processing time depends upon the size of problems, while hardly one minute is required for small problems with less then 500 observations.

After data preparation, the next important task is fitting an appropriate machine learning algorithm to build a predictive model. For this purpose KNIME contains model building nodes for almost all options of machine learning and predictive models including most popular algorithms such as Bayes models, fuzzy rules, fuzzy c-means, k-means, neural networks, decision tree models, hierarchical and the self-organizing tree algorithms, linear and polynomial regression models, support vector machines, and supervised machine learning.

Nonetheless, along with simple statistical analysis and mathematical operations facilities, nodes to perform cross validation and bagging are also available. In addition, to integrate large number of statistical and graphical libraries, R [86] package is supported by KNIME to cover advanced data manipulation and modeling.

Automated QSAR modeling workflow can easily be customized to embed any of the mentioned algorithms. The implemented methodology in the current workflow combines series of R nodes to read data (R Source node), to draw plots (R View node), to train and build model (R Learner and R Predictor nodes) to perform additional tasks by personalized code (R Snippet node). However, major tasks of feature selection by RF (Additional file 1: Figure S3 and Additional file 1: Figure S4) and model building by SVM (Additional file 1: Figure S5) are performed with the help of inter-connected R nodes. Finally, the developed models are saved by model writer node in the user defined directory that can easily be read by model read node to make new predictions.

### Real world cases

#### Data sets description

We tested the proposed QSAR modeling workflow on datasets of different members of protein families. These proteins include glutamate [NMDA] receptor, sigma non-opioid intracellular receptor (Sigma), beta-adrenergic receptor (ADRB), alpha-adrenergic receptor, histamine receptor (HRH), Potassium voltage-gated channel subfamily H member, dopamine (DA-Rs) and serotonin (5-HT) receptors (Table 1). The selection of these thirty different target proteins is independent of any hypothesis. Here, our emphasis is to examine the performance of applied strategy of QSAR modeling to solve diverse issues rather than to produce the best predictive model for each problem. To run the workflow, an initial configuration of “Input Parameter” meta-node is required to set the values of given parameters including number of folds for cross-validation (nfold), target protein name and UniProt accession number, working directory path, fingerprints and associated bioactivity. Hence, to prepare datasets for given problems “Input Parameter” meta-node was configured by providing name and UniProt accession number (Homo sapiens specific) of selected receptors, the associated bioactivity type (Table 1), Morgan fingerprints and “nfold” value was specified to perform tenfold cross validation (nfold = 10).

**Table 1:** Description of selected problems

Uniprot ID	Target protein name	Associated bioactivities (Y)	Total number of observations (N-retrieved)	Total number of observations (N-processed)
Q05586	Glutamate [NMDA] receptor	IC50	512	320
Q99720	Sigma non-opioid intracellular receptor 1 (Sigma1R)	IC50	1895	762
Q99720	Sigma non-opioid intracellular receptor 1 (Sigma1R)	Ki	2584	1465
CHEMBL613288 (Uniprot ID NA.)	Sigma non-opioid intracellular receptor 2 (Sigma2R)	Ki	553	497
P08588	Beta-1 adrenergic receptor (ADRB1)	IC50	1471	599
P07550	Beta-2 adrenergic receptor (ADRB2)	IC50	1424	554
P13945	Beta-3 adrenergic receptor (ADRB3)	EC50	1478	1227
P35348	Alpha-1A adrenergic receptor	Ki	1650	1260
P35368	Alpha-1b adrenergic receptor	Ki	1567	1260
P25100	Alpha-1D adrenergic receptor	Ki	2076	1060
P35367	Histamine H$_1$ receptor (HRH1)	Ki	2239	1222
P25021	Histamine H$_2$ receptor (HRH2)	Ki	1218	385
Q9Y5N1	Histamine H$_3$ receptor (HRH3)	Ki	3799	3101
Q9H3N8	Histamine H$_4$ receptor (HRH4)	Ki	1486	1095
Q12809	Potassium voltage-gated channel subfamily H member 2 (HERG)	Ki	2539	1481
P21728	D(1A) dopamine receptor (DRD1)	Ki	2244	1087
P14416	D(2) dopamine receptor (DRD2)	IC50	1667	725
P35462	D(3) dopamine receptor (DRD3)	IC50	1174	326
P21917	D(4) dopamine receptor (DRD4)	Ki	3409	1900
P21918	D(1B) dopamine receptor (DRD5)	Ki	529	341
P47898	5-Hydroxytryptamine receptor 5A	Ki	382	302
P50406	5-Hydroxytryptamine receptor 6	Ki	4084	2632
P46098	5-Hydroxytryptamine receptor 3A	Ki	517	432
P28222	5-Hydroxytryptamine receptor 1B	Ki	1129	938
P41595	5-Hydroxytryptamine receptor 2B	Ki	2034	1149
P28335	5-Hydroxytryptamine receptor 2C	Ki	3433	2157
P28221	5-Hydroxytryptamine receptor 1D	Ki	1153	973
P08908	5-Hydroxytryptamine receptor 1A	Ki	4008	3244
Q13639	5-Hydroxytryptamine receptor 4	Ki	540	422
P34969	5-Hydroxytryptamine receptor 7	Ki	1753	1438

#### Data preparation and variable scaling

A subset of any data set from ChEMBL Database is passed through the R Snippet node (Data Transformation) (Fig. 4). Variables scaling/transformation is important to standardize the range of independent feature to normalize the highly varying values in raw data for proper functionality of many machine learning algorithms. Recently, ChEMBLdb introduced pChEMBL value, which is an approach to standardize different activity types/values/units. pChEMBL is defined as a negative logarithm of molar ${IC_{\textit{50}}}$, ${XC_{\textit{50}}}$, ${EC_{\textit{50}}}$, ${AC_{\textit{50}}}$, $K_{i}$, ${K_{d}}$ or Potency [42]. Some other methods to normalize widely varying ranges of activity values are also reported in the literature. For example, p$K_{i}$ values are the negative logarithm to base 10 of the equilibrium dissociation constant, which allows an easier comparison of binding affinities. Thus, standard deviations are symmetrical for ${\text{p}}K_{i}$ values but not for $K_{i}$ values [84]. A generic formula was applied to convert values into scaled values (sp(Activity value)) within “Data Transformation” node (Additional file 1: Figure S1) according to the following rules:

5 $$\begin{aligned} {\text{If}}\,{\text{Activity}}\,{\text{value}}&\ge 10{,}000,\quad {\text{sp(Activity}} \,{\text{value)}} = 0 \\{\text{If}}\, 10{,}000& >{\text{Activity}}\,{\text{value}}> 1 ,\\ {\text{sp(Activity}}\, {\text{value)}}&= \frac{(4-\log 10(Activity\; value ))}{4}\\ {\text{If}}\, 1&\ge {\text{Activity}}\,{\text{value}},\quad {\text{sp(Activity value)}} = 1\end{aligned}$$

where sp(Activity value) represents the scaled activity value.

Finally, after normalization of response variables (bioactivities) data is divided by random sampling into 75% training set and 25% independent validation set that will not be used in any training process (Fig. 4).

#### Data set modelability measure

As stated, before the modeling phase of the thirty selected problems, the “modelability index” (MODI) is calculated [53] (Additional file 1: Figure S1 and Table S1). MODI requires that the activities of compounds in all data sets and their distribution in the descriptor space (predictors) must range in the interval [0, 1]. Biological activities were scaled according to Eq. 5, while descriptors were processed using a simple [0, 1] scaling (Eq. 6).

6 $$x' = \frac{x-min(x)}{max(x)-min(x)}$$

where *x* is the original descriptor and $x'$ is the scaled result of that variable.

#### Feature ranking by Random Forest

Data sets of all descriptors (descriptors and fingerprints) was used to consider high dimensional data sets for unbiased implementation of developed workflow to build an robust model based on best relevant features from highly redundant data.

This framework identify the most important features the ones that are responsible for the relevant molecular activity. Feature selection is a crucial step to reduce computation time and storage, improve model interpretability, understanding, performance, and remove irrelevant features (noisy data) to avoid over fitting [87]. Hence, we followed a strong method of RF based feature selection with a particular emphasis to generate more reliable, predictable, and generalized QSAR models [45]. QSAR modeling workflow finds the ranked ordered list of variables (descriptors and fingerprints) according to both scaled ((VI)1) and unscaled ((VI)2) importance scores (Additional file 1: Figure S3 and Figure S4).

Due to the stochastic nature of the RF algorithm, nfold cross validation was performed to fit RF models, and the importance of variables was recorded for each run. In the end, variables were ranked by sorting average variable importance scores in descending order. The process of features ranking is performed by two kinds of meta-nodes including “Model Validation” and “mean(%MSE) Calculator” (Additional file 1: Figure S3 and Figure S4). Hence, the output of these two meta-nodes is a processed input data rearranged by two kinds of variable rankings methods, first by scaled variable importance based ranked order, and second by unscaled importance based variables ranking.

#### Stepwise estimation models and feature selection

The produced ordered training data with more relevant to less important variables was further processed by meta-node “Build Model by Adding Ranked Variables”, which firstly splits data into training and test set and introduces each ranked variable into a new SVMs fitted models (Additional file 1: Figure S5). Each new model is validated by test set, and the statistical results of these stepwise estimation models are recorded to find the best set of features with minimum predictive error (RMSE). The results of the selected features based models (SF-models) of all target proteins clearly indicate large reduction of the total number of features (F) into more relevant features (SF) in all data sets. In the given problems, the maximum reduction of the features is 1037–9 variables ranked by scaled importance approach and 1079–29 variables in the case of unscaled importance. Similarly, the minimum reduction is 1134–470 variables and 1132–432 variables by scaled and unscaled importance methods respectively. Hence, on average applied methodology of feature selection performs adequate dimensionality reduction that is an important task to improve the quality of the predictive model.

#### Model results

After selecting the predictive model with best set of features (SF-model), the model’s final assessment was performed using of the IVS. External validation is a critical step to make sure unbiased evaluation of developed model [20, 22, 44]. The IVS considered for external validation was never used in feature reduction and model training processes . On average, the difference between predictive performance of internally and externally validated SF-models is not large with optimally fitted models (Table 2). SF-models of three receptors including Sigma1R (bioactivity dataset of *IC*50), 5-HT2B and 5-HT4 showed poor generalization due to over-fitting in both methods of feature selection. In the other cases some SF-models like Sigma1R (bioactivity data set of $K_{i}$), 5-HT1A, 5-HT3A, 5-HT5A, 5-HT1D, ADRB1, DRD4 and DRD5 performed even better for external predictions.

**Table 2:** QSAR models based on all descriptors (RDKit descriptors and Morgan fingerprints) datasets

Target protein name	Total number of observations (N-processed)		Total number of features (F)	Feature selection by scaled variables importance (VI)1					Feature selection by unscaled variables importance (VI)2				
	Training set	IVS		Selected features (SF)	SF-model (test set)		Final model (SF-model (IVS))		Selected features (SF)	SF-model (test set)		Final model (SF-model (IVS))	
					PVE	RMSE	PVE	RMSE		PVE	RMSE	PVE	RMSE
Glutamate [NMDA] receptor	240	80	949	120	0.78	0.12	0.69	0.17	120	0.79	0.12	0.73	0.16
Sigma non-opioid intracellular receptor 1 (Sigma1R)	572	190	1079	220	0.68	0.15	0.47	0.19	29	0.62	0.16	0.40	0.20
Sigma non-opioid intracellular receptor 1 (Sigma1R)	1099	366	1117	111	0.64	0.17	0.60	0.18	116	0.59	0.17	0.61	0.17
Sigma non-opioid intracellular receptor 2 (Sigma2R)	373	124	875	201	0.71	0.11	0.57	0.14	234	0.66	0.13	0.61	0.14
Beta-1 adrenergic receptor (ADRB1)	450	149	1040	150	0.70	0.14	0.72	0.13	180	0.80	0.12	0.71	0.13
Beta-2 adrenergic receptor (ADRB2)	416	138	1032	133	0.76	0.13	0.70	0.16	76	0.75	0.13	0.69	0.16
Beta-3 adrenergic receptor (ADRB3)	921	306	1093	310	0.64	0.15	0.56	0.17	170	0.57	0.19	0.55	0.18
Alpha-1A adrenergic receptor	945	315	1108	206	0.69	0.16	0.67	0.18	170	0.73	0.16	0.66	0.18
Alpha-1b adrenergic receptor	945	315	1106	275	0.71	0.15	0.65	0.15	115	0.69	0.15	0.62	0.16
Alpha-1D adrenergic receptor	795	265	1109	270	0.69	0.16	0.65	0.17	370	0.68	0.16	0.66	0.17
Histamine H$_1$ receptor (HRH1)	917	305	1116	76	0.79	0.15	0.72	0.17	237	0.79	0.14	0.76	0.16
Histamine H$_2$ receptor (HRH2)	289	96	1037	9	0.30	0.11	0.32	0.13	180	0.62	0.07	0.33	0.13
Histamine H$_3$ receptor (HRH3)	2326	775	1134	397	0.62	0.16	0.63	0.16	282	0.66	0.16	0.63	0.16
Histamine H$_4$ receptor (HRH4)	822	273	1075	123	0.63	0.18	0.56	0.18	330	0.63	0.17	0.55	0.18
Potassium voltage-gated channel subfamily H member 2 (HERG)	1111	370	1132	120	0.69	0.12	0.54	0.15	160	0.64	0.12	0.55	0.15
D(1A) dopamine receptor (DRD1)	816	271	1118	118	0.73	0.15	0.68	0.17	219	0.75	0.15	0.70	0.16
D(2) dopamine receptor (DRD2)	544	181	1092	91	0.66	0.16	0.63	0.18	150	0.71	0.16	0.62	0.19
D(3) dopamine receptor (DRD3)	245	81	1054	36	0.58	0.21	0.58	0.19	195	0.66	0.18	0.61	0.18
D(4) dopamine receptor (DRD4)	1425	475	1124	368	0.60	0.18	0.63	0.17	395	0.60	0.18	0.62	0.17
D(1B) dopamine receptor (DRD5)	256	85	957	135	0.68	0.18	0.76	0.15	142	0.75	0.17	0.77	0.15
5-Hydroxytryptamine receptor 5A	227	75	980	140	0.83	0.13	0.87	0.12	38	0.81	0.14	0.84	0.13
5-Hydroxytryptamine receptor 6	1974	658	1132	320	0.72	0.15	0.68	0.16	432	0.69	0.17	0.67	0.16
5-Hydroxytryptamine receptor 3A	324	108	1045	150	0.69	0.19	0.71	0.19	230	0.62	0.21	0.71	0.19
5-Hydroxytryptamine receptor 1B	704	234	1103	255	0.79	0.15	0.75	0.16	145	0.79	0.15	0.76	0.15
5-Hydroxytryptamine receptor 2B	862	287	1130	101	0.51	0.18	0.37	0.19	110	0.57	0.15	0.39	0.19
5-Hydroxytryptamine receptor 2C	1618	539	1135	263	0.67	0.16	0.62	0.18	244	0.64	0.18	0.62	0.17
5-Hydroxytryptamine receptor 1D	730	243	1112	120	0.82	0.15	0.76	0.18	250	0.76	0.19	0.77	0.18
5-Hydroxytryptamine receptor 1A	2433	811	1134	470	0.61	0.19	0.65	0.17	360	0.59	0.19	0.66	0.17
5-Hydroxytryptamine receptor 4	317	105	948	203	0.80	0.16	0.66	0.22	280	0.83	0.15	0.71	0.20
5-Hydroxytryptamine receptor 7	1079	359	1122	210	0.65	0.16	0.59	0.18	290	0.66	0.16	0.61	0.17

To validate the efficiency of the implemented methodology, a model was also developed without feature selection (full-model). The external validation score of full-model is also calculated to compare the performance with final predictive model with selected features (SF-model). The comparison of the performance of externally validated full model and externally validated final SF-model clearly confirms the effectiveness of the feature selection method. The results from all thirty different data sets show a significant increase in predictive power (PVE) and reduction in prediction error (RMSE) by removing the noisy data and considering the most relevant features (Table 3).

**Table 3:** Comparison of performance of QSAR models (with and without feature selection)

Target protein name	Total number of observations (N-processed)		Total number of features (F)	PVE (IVS)			RMSE (IVS)		
	Training set	IVS		Full model without feature delection	Final model with feature selection		Full model without feature selection	Final model with feature selection	
				Full-model	SF-model (VI)1	SF-model (VI)2	Full-model	SF-model (VI)1	SF-model (VI)2
Glutamate [NMDA] receptor	240	80	949	0.30	0.69	0.73	0.25	0.17	0.16
Sigma non-opioid intracellular receptor 1 (Sigma1R)	572	190	1079	0.31	0.47	0.40	0.21	0.19	0.20
Sigma non-opioid intracellular receptor 1 (Sigma1R)	1099	366	1117	0.45	0.60	0.61	0.21	0.18	0.17
Sigma non-opioid intracellular receptor 2 (Sigma2R)	373	124	875	0.46	0.57	0.61	0.16	0.14	0.14
Beta-1 adrenergic receptor (ADRB1)	450	149	1040	0.41	0.72	0.71	0.19	0.13	0.13
Beta-2 adrenergic receptor (ADRB2)	416	138	1032	0.46	0.70	0.69	0.21	0.16	0.16
Beta-3 adrenergic receptor (ADRB3)	921	306	1093	0.37	0.56	0.55	0.21	0.17	0.18
Alpha-1A adrenergic receptor	945	315	1108	0.53	0.67	0.66	0.21	0.18	0.18
Alpha-1b adrenergic receptor	945	315	1106	0.48	0.65	0.62	0.18	0.15	0.16
Alpha-1D adrenergic receptor	795	265	1109	0.47	0.65	0.66	0.21	0.17	0.17
Histamine H$_1$ receptor (HRH1)	917	305	1116	0.59	0.72	0.76	0.21	0.17	0.16
Histamine H$_2$ receptor (HRH2)	289	96	1037	0.13	0.32	0.33	0.14	0.13	0.13
Histamine H$_3$ receptor (HRH3)	2326	775	1134	0.46	0.63	0.63	0.19	0.16	0.16
Histamine H$_4$ receptor (HRH4)	822	273	1075	0.34	0.56	0.55	0.22	0.18	0.18
Potassium voltage-gated channel subfamily H member 2 (HERG)	1111	370	1132	0.42	0.54	0.55	0.17	0.15	0.15
D(1A) dopamine receptor (DRD1)	816	271	1118	0.50	0.68	0.70	0.21	0.17	0.16
D(2) dopamine receptor (DRD2)	544	181	1092	0.51	0.63	0.62	0.21	0.18	0.19
D(3) dopamine receptor (DRD3)	245	81	1054	0.32	0.58	0.61	0.24	0.19	0.18
D(4) dopamine receptor (DRD4)	1425	475	1124	0.47	0.63	0.62	0.20	0.17	0.17
D(1B) dopamine receptor (DRD5)	256	85	957	0.56	0.76	0.77	0.20	0.15	0.15
5-Hydroxytryptamine receptor 5A	227	75	980	0.58	0.87	0.84	0.22	0.12	0.13
5-Hydroxytryptamine receptor 6	1974	658	1132	0.48	0.68	0.67	0.20	0.16	0.16
5-Hydroxytryptamine receptor 3A	324	108	1045	0.41	0.71	0.71	0.27	0.19	0.19
5-Hydroxytryptamine receptor 1B	704	234	1103	0.45	0.75	0.76	0.23	0.16	0.15
5-Hydroxytryptamine receptor 2B	862	287	1130	0.31	0.37	0.39	0.20	0.19	0.19
5-Hydroxytryptamine receptor 2C	1618	539	1135	0.48	0.62	0.62	0.21	0.18	0.17
5-Hydroxytryptamine receptor 1D	730	243	1112	0.49	0.76	0.77	0.24	0.18	0.18
5-Hydroxytryptamine receptor 1A	2433	811	1134	0.43	0.65	0.66	0.21	0.17	0.17
5-Hydroxytryptamine receptor 4	317	105	948	0.35	0.66	0.71	0.26	0.22	0.20
5-Hydroxytryptamine receptor 7	1079	359	1122	0.43	0.59	0.61	0.21	0.18	0.17

In the developed QSAR models of selected problems, the PVE score of the full-model ranges 0.13–0.59 while in the SF-model PVE ranges between 0.32–0.87 and 0.33–0.84 from scaled importance ((VI)1) and unscaled importance ((VI)2) methods respectively. However, an average PVE increase in both methods, ((VI)1) and ((VI)2) is almost 49% of the PVE of the full-model. The number of features in SF-models ranges between 0.0079–16% of the total number of processed features considered in full models, which contain 1135 variables. The average reduction in the number of features is 83% of the total number. Moreover, error analysis of all predictive models shows an average RMSE of the full-model is 0.21 and in the case of SF-model the average RMSE is 0.17 in both methods. Hence. an average error decrease is 19% of the RMSE of the full-model. The large improvement of SF-models predictive performance and decrease in error rate exhibit the strength of unbiased methodology followed in automated QSAR modeling workflow.

All intermediate results can be visualized by interactive tables and graphical outputs from data visualization layers (Additional file 1: Figure S1). After completion of the QSAR model building workflow, outputs of each task are saved in the user’s defined working directory (Additional file 1: Figure S6). The availability of these intermediate data in the end of each task is useful to restore historical predictions and the given processed data with filtered features can further be used in any other application.

#### Model applicability domain analysis

For all thirty problems, feature selection and model development was carried out using the training set; however, model applicability to external compounds depends on the structural similarity between the chemicals in the IVS and the training set molecules. Model predictability is considered more reliable if the IVS chemicals fall within the AD. We used a KNIME node “Domain-Similarity” (Additional file 1: Figure S1) [82, 88] to analyze the AD of the models developed by the presented workflow. “Domain-Similarity” node uses similarity measurements to define the AD using Euclidean distances among all training compounds and the test or IVS compounds. The prediction may be unreliable if the distance of an external set compound to its nearest neighbor in the training set is higher than defined AD (out of AD).

In majority of the thirty selected problems compounds within the IVS were inside the AD, with the exception of six problems where some instances were outside the AD. These are the D(1A) dopamine receptor (3 molecules outside the AD), D(2) dopamine receptor (2 molecules), D(3) dopamine receptor (2 molecules), Sigma non-opioid intracellular receptor 1 with activity $K_{i}$ (1 molecule), HRH2 (1 molecule), and 5-hydroxytryptamine receptor 1D (1 molecule). As the IVS should be a data set not controlled by the modellers, this QSAR modeling workflow does not remove these molecules and the decision is left to the users on how to handle the more prediction-error prone instances of the IVS.

#### Predictive performance comparison with published QSAR model

In the above analysis of the selected thirty problems, “Fully Automated” mode was tested where all processes from data retrieval to model building are completely automated (Fig. 3). We further used “Customized” mode, of the workflow (Fig. 3), to demonstrate the efficiency of implemented methodology in the developed automated QSAR model by comparing its performance to the published solutions of scientific problems. For this purpose, we selected one very recent example on antiviral binding affinity data for non-nucleoside analogue reverse-transcriptase inhibitors (NNRTIs) from the QsarDB repository [89]. The same training (31 molecules) and external validation (8 molecules) datasets of chemical compounds with their corresponding scaled bioactivity (${pK_{i}}$) were taken from the published work [90] for model building in this workflow. The curated dataset of NNRTIs with the 39 ligands in SMILES format and their computed ${pK_{i}}$ was submitted in “Customized” mode option 1 (Fig. 3). As $K_{i}$ values were already scaled [90], so we skipped the “Data Transformation” node and adjusted the data partitioning node for the simple division of reported 31 training and 8 IVS molecules (Fig. 4). RDKit descriptors and fingerprints were computed automatically for this given input dataset of NNRTIs. MODI scores for the first three options of fingerprints (Morgan, FeatMorgan, AtomPair) in the “Input Parameter” meta-node (Fig. 4) were lower than the threshold (${\text {MODI}} >0.45$). Thus, we skipped these 3 fingerprints and continued the modeling process using RDKit descriptors and torsion fingerprints for which MODI score was greater than the threshold (for K3, MODI = 0.46 and for K5, MODI = 0.48).

Performance of automated QSAR modeling workflow based SF-models in antiviral binding affinity prediction on external validation set or IVS for NNRTIs was markedly better in both options (scaled and unscaled variable importance) of feature selection than the published [90] QSAR model. The PVE score of the SF-model((VI)1) is 0.81 and for SF-model((VI)2) is 0.82 while the published solution showed 0.725 scores of the squared coefficient of correlation (R$^{2}$) for the same IVS. In the same way, the RMSE score of the SF-model((VI)1) is 0.34 and for SF-model((VI)2) is 0.33 while the published solution showed 0.2230 (RMSE= 0.47) score of squared standard error of the regression (S$^{2}$) for the same IVS. All the molecules of the IVS were found within the AD; thus predictions can be considered reliable.

## Discussion

In the current work, an extendable platform was designed that can be used as a QSAR modeling pipeline to get an optimized predictive model. The performance of the presented automated QSAR modeling workflow was assessed for thirty different data sets of size ranging from 300 to 3200 molecules and the features set of 1141 descriptors (RDKit descriptors and fingerprints). We have further compared the results obtained from our workflow with a published QSAR modeling problem and the results obtained were significantly better than the original authors efforts, even though the approach followed was mostly unsupervised.

**Fig. 5:**
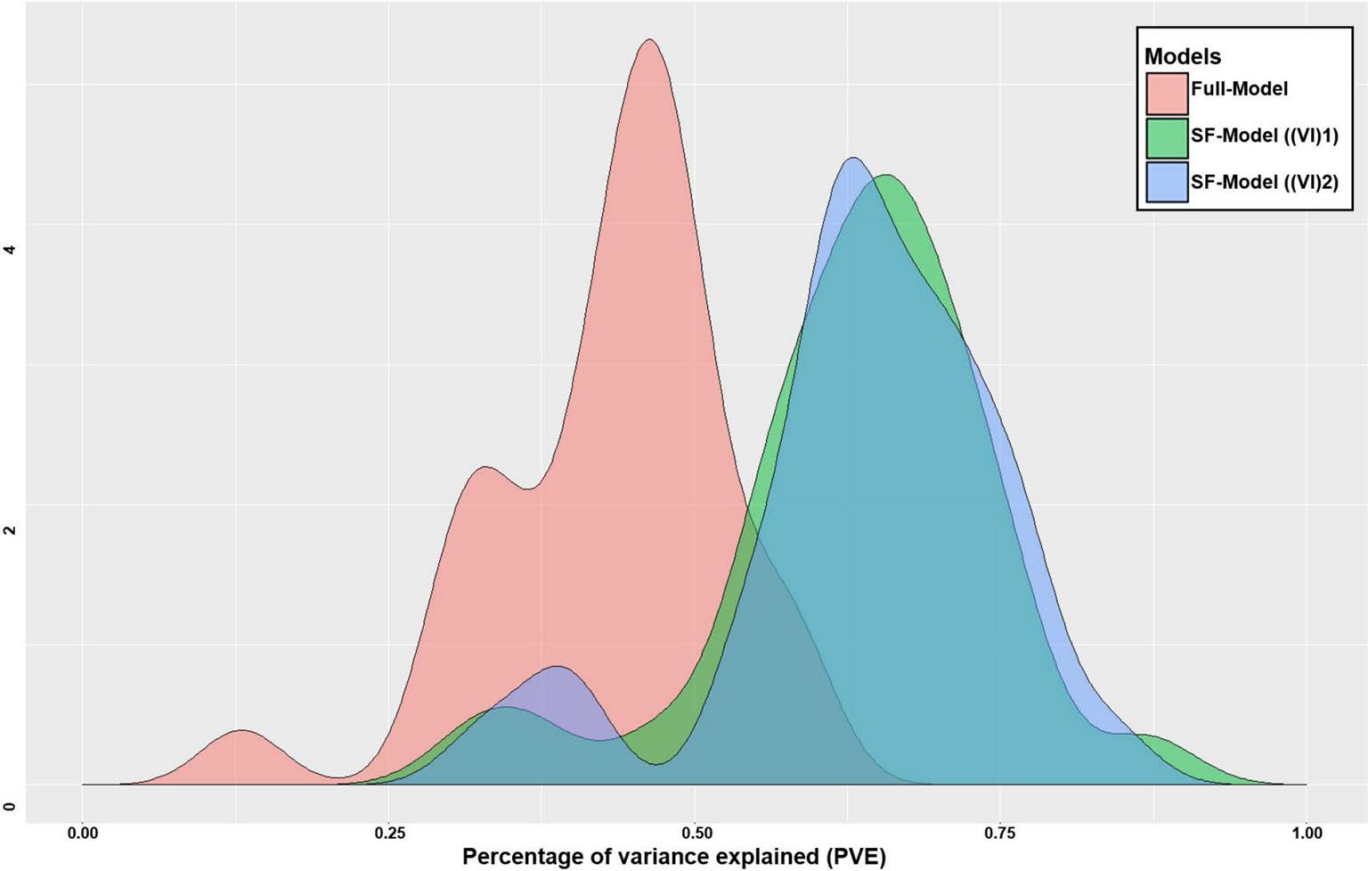
Comparison of models with and without feature selection. Pink color represents the full-model without feature selection [with all variables (F)], green color is for SF-model ((VI)1) contains predefined set of features (SF) identified by scaled permutation importance, and blue color represents SF-model ((VI)2) having selected features (SF) by unscaled variable importance measure

Comparison of all constructed full-models and SF-models revealed improved predictive power with a small set of biologically relevant variables (Fig. 5). Hence, feature selection methodology was found efficient to deal with high dimensional data by selecting adequate features for each problem to predict a certain property with a good performance, less computational/time cost. For regression problems, compelling evidences exists for the robustness of RF unscaled variable importance measure VI(Xj) because of its statistical properties [72, 72, 73]. Consistent with literature, overall performance of selected sub-set of variables by RF unscaled importance measure ((VI)2) was better than scaled importance measure ((VI)1).

**Fig. 6:**
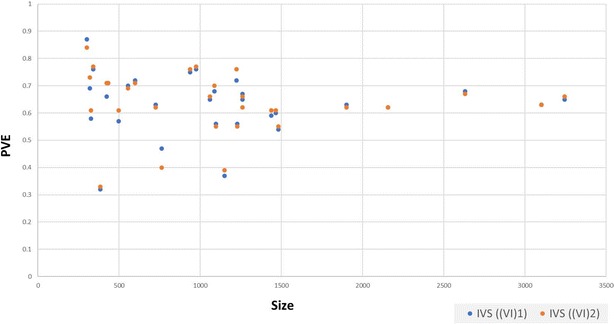
Size of the problems and predictive power of fitted models. Blue dots represent externally validated models with feature selection by scaled importance, and golden yellow color denotes externally validated models with feature selection by unscaled importance measure

To explore the role of the training data sets size in determining the performance of predictive models, PVE for each model was compared with data set size (Fig. 6). Models trained with data sets less then 1500 molecules showed quite diverse predictive performance. The dat set size of the best performing model of the receptor 5-HT5A with PVE value 0.87 is 302 molecules and least performing model of the receptor HRH2 with PVE value 0.32 has 385 molecules. The models performance was stable in larger data sized problems. Possible reasons for these variations in performance is may be the complex nature of the problem and the size limitations [44]. Hence, availability of more data may help to find real trends in data with a satisfactory solution.

**Fig. 7:**
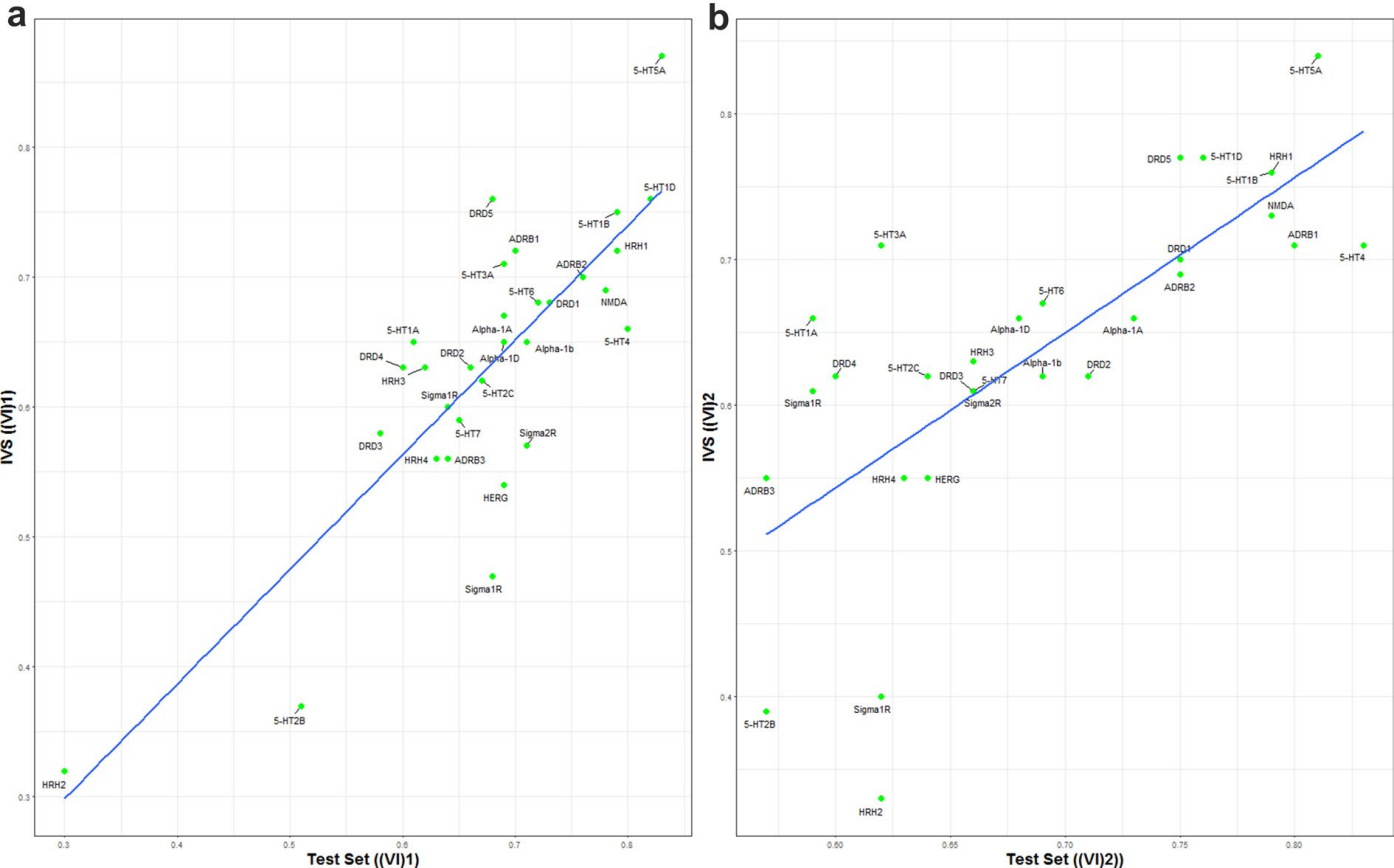
Models over-fitting analysis. Models with a predefined set of features identified by scaled variable importance (**a**) and unscaled variable importance (**b**)

In regression modeling, one of the most critical problem is over-fitting of a model which results into poor generalization and reduced performance on unseen data. One widely accepted measure for testing over-fitting is to observe performance over independent validation data set [4, 22]. Hence, SF-model’s final assessment was performed using of the independent validation set (IVS). The internal (test set) and external (IVS) prediction results of the SF-models were compared to identify the over-fitted models (Fig. 7) in both methods of feature selection like the scaled (Fig. 7a) and unscaled importance (Fig. 7b). In both feature selection methods, none of both is completely superior to the other one. For example, problem Histamine H$_2$ receptor (HRH2) is a worst generalized model constructed by unscaled importance based feature selection, but was optimally fitted by the scaled importance based set of features. Hence, our focus was on the problems that were failed in both feature selection methods. Out of thirty problems, three models were found over-fitted in both methods.

Worst cases include 5-hydroxytryptamine receptor 2B (5-HT2B), 5-hydroxytryptamine receptor 4 (5-HT4) and Sigma non-opioid intracellular receptor 1 (Sigma1R) that are over-fitted in both variable selection processes. Comparison of experimental and predicted activity values was carried out to analyze poor prediction of particular activity value points (Additional file 1: Figure S7). The over-fitted models were unable to accurately predict the response variable at extreme values and large errors were observed near the upper and lower extreme of the experimental range. These mispredictions may result from data sets with very few measured instances with values near the experimental range. However, insufficient patterns of predictors may reduce the model coverage and lead to poor generalization [44].

**Fig. 8:**
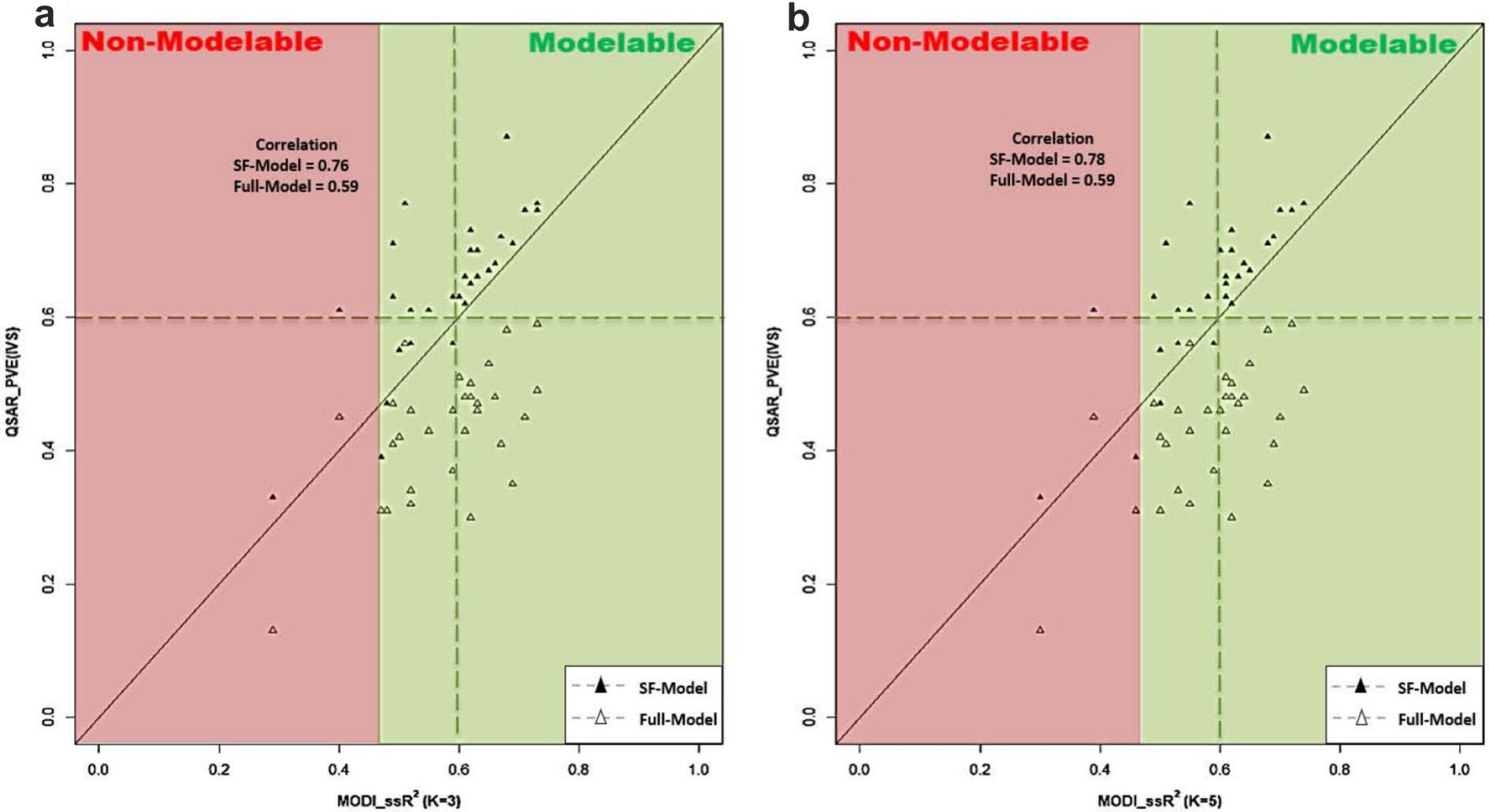
$MODI\_ssR^{2}$ versus QSAR_PVE for 30 datasets. K is the number of nearest neighbors. **a** K = 3 and **b** K = 5. QSAR_PVE(IVS) is PVE score of externally validated models without feature selection (Full-model) and with selected features (SF-model). High correlation with SF-models QSAR_PVE suggests $MODI\_ssR^{2}$ is good modelability criteria. Weaker correlation between Full-model QSAR_PVE and $MODI\_ssR^{2}$ emphasize the importance of feature selection to obtain actual and reliable predictive performance of QSAR model

In the end, PVE scores (QSAR_PVE(IVS)) of full-models and final SF-models were compared with their corresponding $MODI\_ssR^{2}$ scores (Fig. 8 and Additional file 1: Table S1). Results showed significant correlation between the PVE for the IVS in SF-models and $MODI\_ssR^{2}$ (correlation = 0.76 for $MODI\_ssR^{2}$ with K = 3 and correlation = 0.78 for $MODI\_ssR^{2}$ with K = 5) (Fig. 8a, b). This is consistent with the published work [53], which suggests that the $MODI\_ssR^{2}$ score should be $\ge \,0.46$ for 3 nearest neighbors and $\ge 0.47$ for 5 nearest neighbors.The correlation between the full-models PVE and $MODI\_ssR^{2}$ was not as significant. This weaker correlation was expected as full-models may contain irrelevant and highly correlated variables which directly influence the models predictive power by causing them to over fit the training sets. Hence, the implemented feature selection approach has an efficient role for achieving robust models with reliable predictive performance.

## Conclusion

The developed QSAR modeling workflow is a fully automated QSAR pipeline to assist all users including those are not expert in machine learning and have less knowledge of available data. Creation of an optimal predictive model demands many critical and time-consuming steps, including data collection and processing, appropriate data representation (descriptors and fingerprints calculation), evaluation of the data set modelability, best predictors selection, machine learning models fitting and validation. QSAR modeling workflow completely automates the laborious and iterative process of modeling to tackle different problems. Following are the key advantages of proposed QSAR modeling workflow:

- It automatically fetches high-quality compounds data set from continuously improving and growing curated databases (e.g. ChEMBL). Hence, the potential of direct access of the online data sets enables to this fully automated framework a widely used platform for QSAR model building.
- Important aspects of the data processing by selecting only the bioactivity type of interest, dealing with duplicates, removing missing data and salt groups, descriptors calculation, and data normalization are handled in a very flexible and consistent manner.
- Prior estimate of data set modelability can reduce modelers efforts by focusing in the most promising problems or identifying the challenging ones that may require more data, more descriptor variability or different strategies.
- Best practice feature selection and an exhaustive validation procedure are followed in the presented workflow in order to ensure minimal bias in model development and evaluation. The analysis of the obtained results of thirty different target–drug interaction predictive models concludes that the developed feature selection methodology performs consistently well for high-dimensional data by removing 62–99% redundant data. This large reduction of irrelevant variables minimizes the computational/time cost, improves the predictive power of model and provides a better understanding of the underlying relationship between the property of interest and the relevant features.
- The automated QSAR modeling framework is not a black-box prediction system, rather it is an extensible and highly customizable tool to develop the robust predictive models and provide the output of all modeling task for the diverse application and reproduction of historical predictions. Moreover, it ensures that the same protocol is used for updating models with new molecules as they become available.
- It is worth mentioning that the generated workflow feeds the selected feature-matrix to SVM models but these variables can be used as input for any other non-linear machine learning method which can be easily implemented in the framework.

In conclusion, with the above mentioned adopted features of the presented open source automated QSAR modeling framework, it is hoped to guarantee that the most important aspects of QSAR modeling are addressed and consistently applied. This framework has been tested against thirty data sets, some very difficult, and generally as produced robust results; this has been achieved without any need of users thorough understanding of data, computer programming and/or machine-learning knowledge and complex parameterization to customize the complex modeling algorithms and procedures.

## Additional files

**Additional file 1.** **Figure S1.** KNIME overview of automated QSAR modeling workflow. **Figure S2.** ChEMBLdb meta node. **Figure S3.** Nfold cross validation meta node. **Figure S4.** MeanMSE meta node. **Figure S5.** Stepwise estimation models meta node. **Figure S6.** Output files generated by automated QSAR modeling workflow. **Figure S7.** Mispredictions of over-fitted models. **Table S1.** Data modelability measure (*MODI_ssR*^2^) versus QSAR_PVE for 30 datasets.

**Additional file 2.** QSAR modeling workflow source file. A zipped file of the QSAR modeling workflow is provided.
