# Supplementary figures and images for: An automated framework for QSAR model building
Source: J Cheminform. 2018 Jan 16;10:1. doi: 10.1186/s13321-017-0256-5 (PMC5770354; doi:10.1186/s13321-017-0256-5)

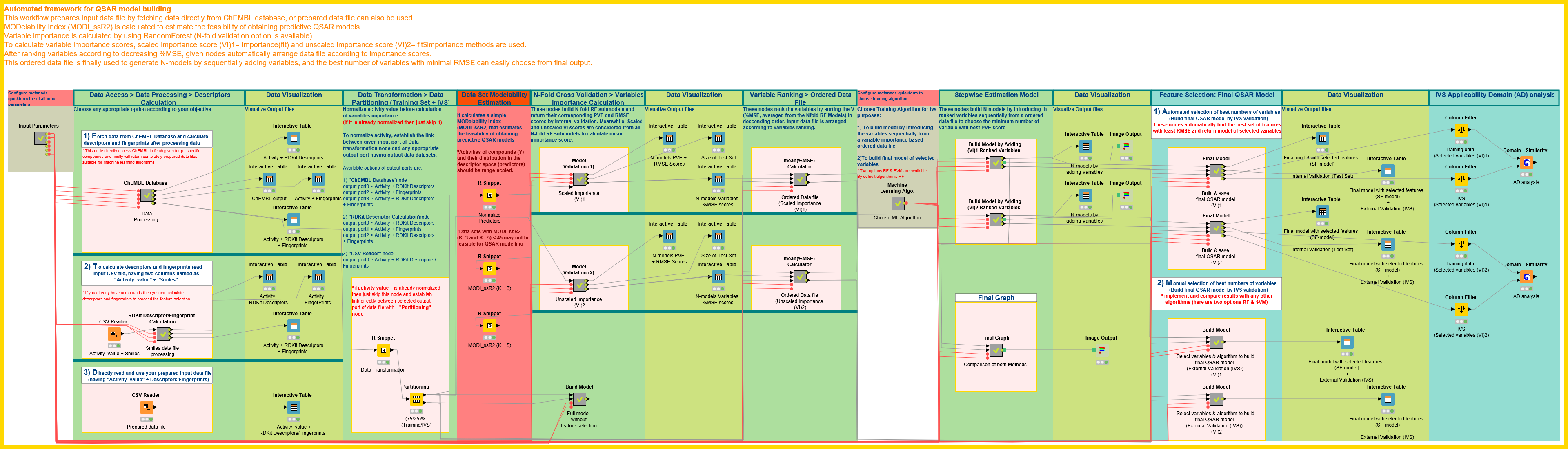

Supplement: Supplementary file 1 — Additional file 1. Figure S1. KNIME overview of automated QSAR modeling workflow. Figure S2. ChEMBLdb meta node. Figure S3. Nfold cross validation meta node. Figure S4. MeanMSE meta node. Figure S5. Stepwise estimation models meta node. Figure S6. Output files generated by automated QSAR modeling workflow. Figure S7. Mispredictions of over-fitted models. Table S1. Data modelability measure (MODI_ssR2) versus QSAR_PVE for 30 datasets. [file 13321_2017_256_MOESM1_ESM.zip › Additional file 1/Figure S1.png]

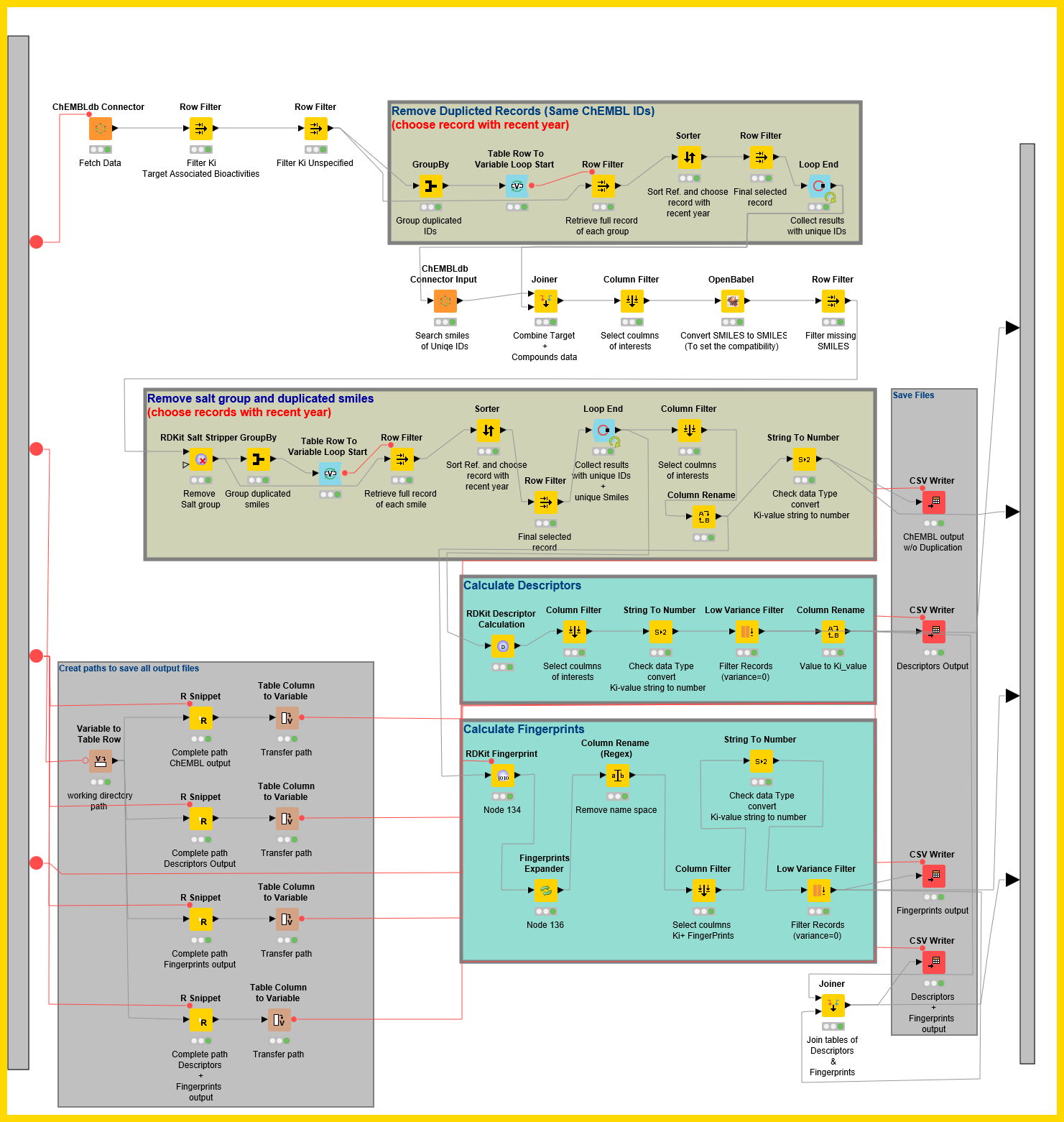

Supplement: Supplementary file 1 — Additional file 1. Figure S1. KNIME overview of automated QSAR modeling workflow. Figure S2. ChEMBLdb meta node. Figure S3. Nfold cross validation meta node. Figure S4. MeanMSE meta node. Figure S5. Stepwise estimation models meta node. Figure S6. Output files generated by automated QSAR modeling workflow. Figure S7. Mispredictions of over-fitted models. Table S1. Data modelability measure (MODI_ssR2) versus QSAR_PVE for 30 datasets. [file 13321_2017_256_MOESM1_ESM.zip › Additional file 1/Figure S2.png]

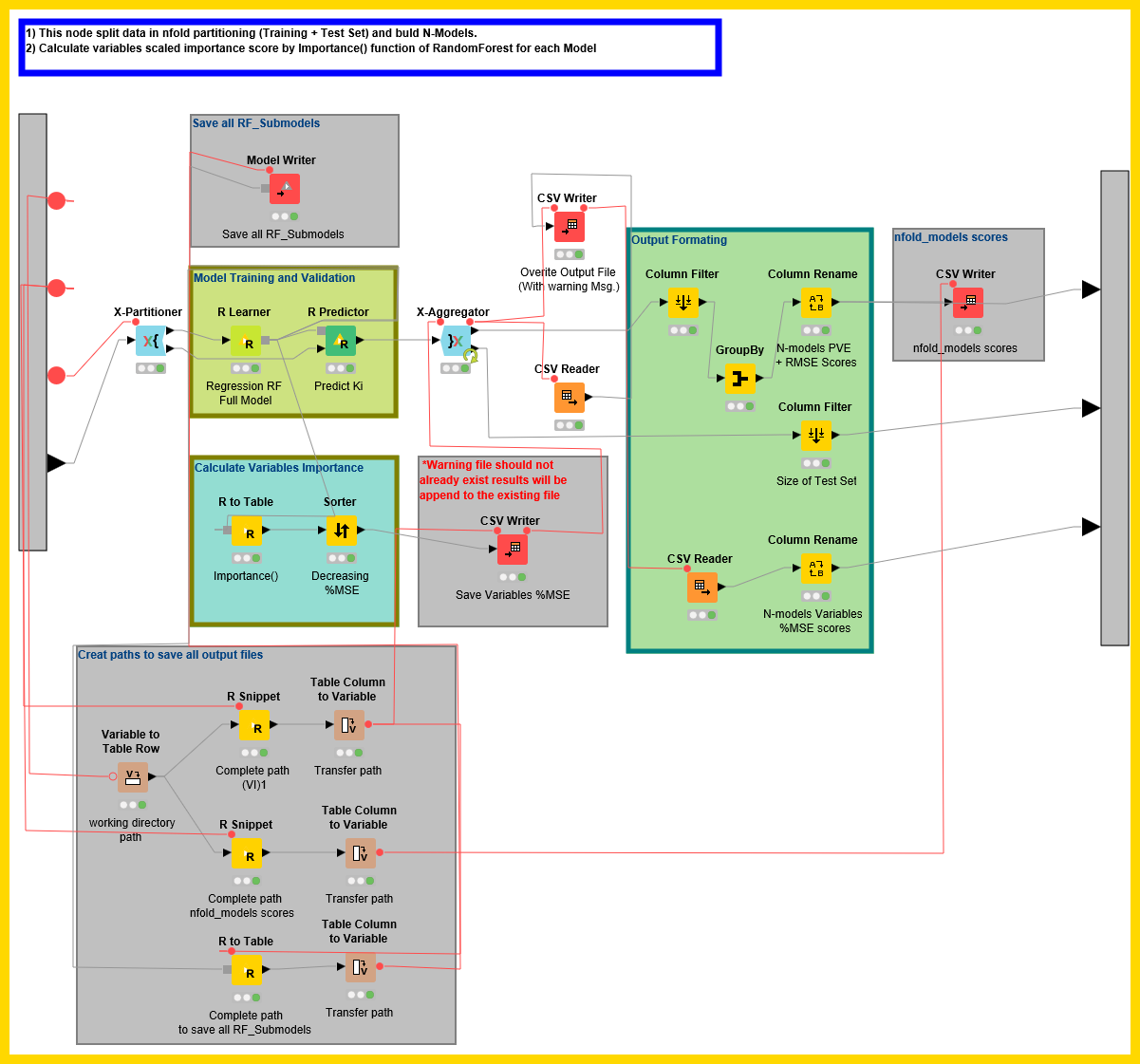

Supplement: Supplementary file 1 — Additional file 1. Figure S1. KNIME overview of automated QSAR modeling workflow. Figure S2. ChEMBLdb meta node. Figure S3. Nfold cross validation meta node. Figure S4. MeanMSE meta node. Figure S5. Stepwise estimation models meta node. Figure S6. Output files generated by automated QSAR modeling workflow. Figure S7. Mispredictions of over-fitted models. Table S1. Data modelability measure (MODI_ssR2) versus QSAR_PVE for 30 datasets. [file 13321_2017_256_MOESM1_ESM.zip › Additional file 1/Figure S3.png]

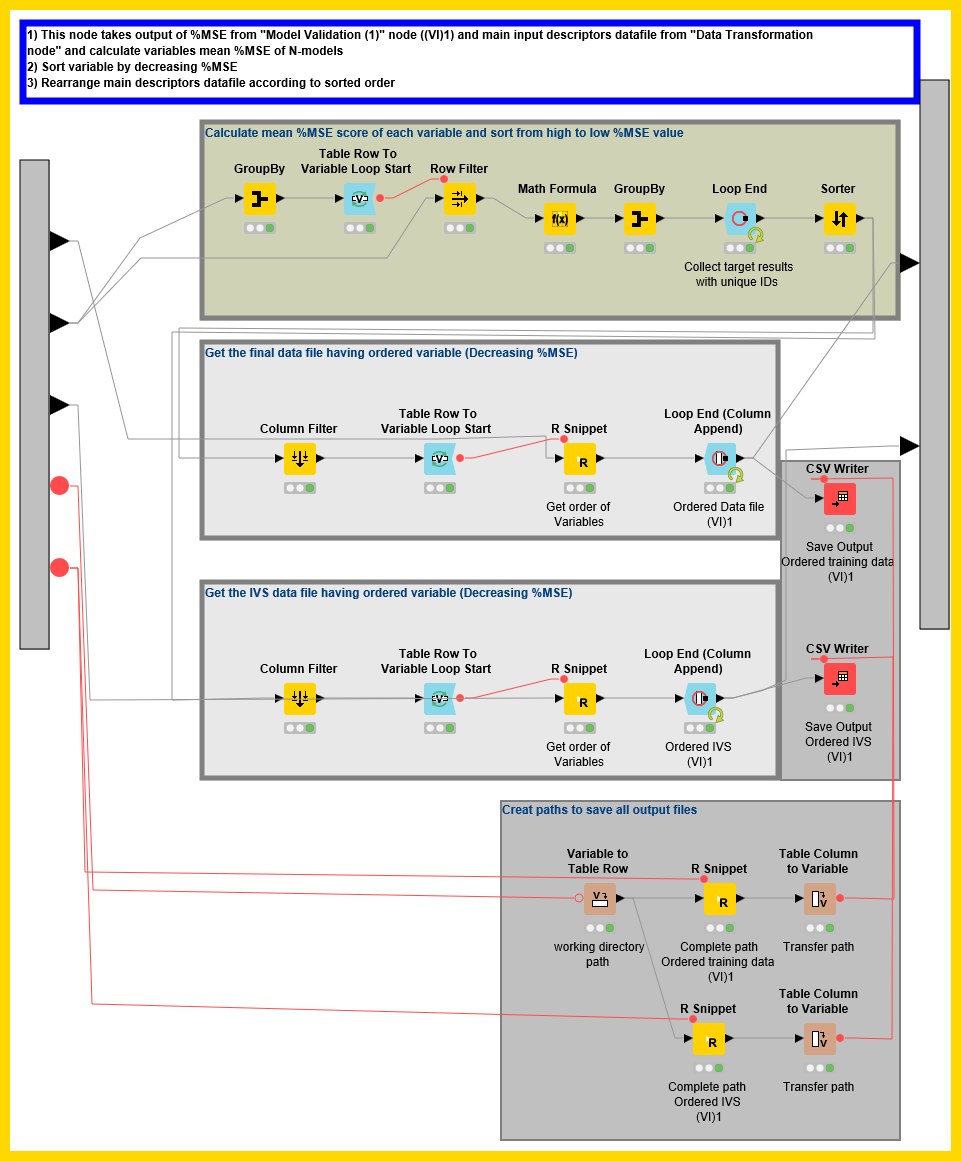

Supplement: Supplementary file 1 — Additional file 1. Figure S1. KNIME overview of automated QSAR modeling workflow. Figure S2. ChEMBLdb meta node. Figure S3. Nfold cross validation meta node. Figure S4. MeanMSE meta node. Figure S5. Stepwise estimation models meta node. Figure S6. Output files generated by automated QSAR modeling workflow. Figure S7. Mispredictions of over-fitted models. Table S1. Data modelability measure (MODI_ssR2) versus QSAR_PVE for 30 datasets. [file 13321_2017_256_MOESM1_ESM.zip › Additional file 1/Figure S4.png]

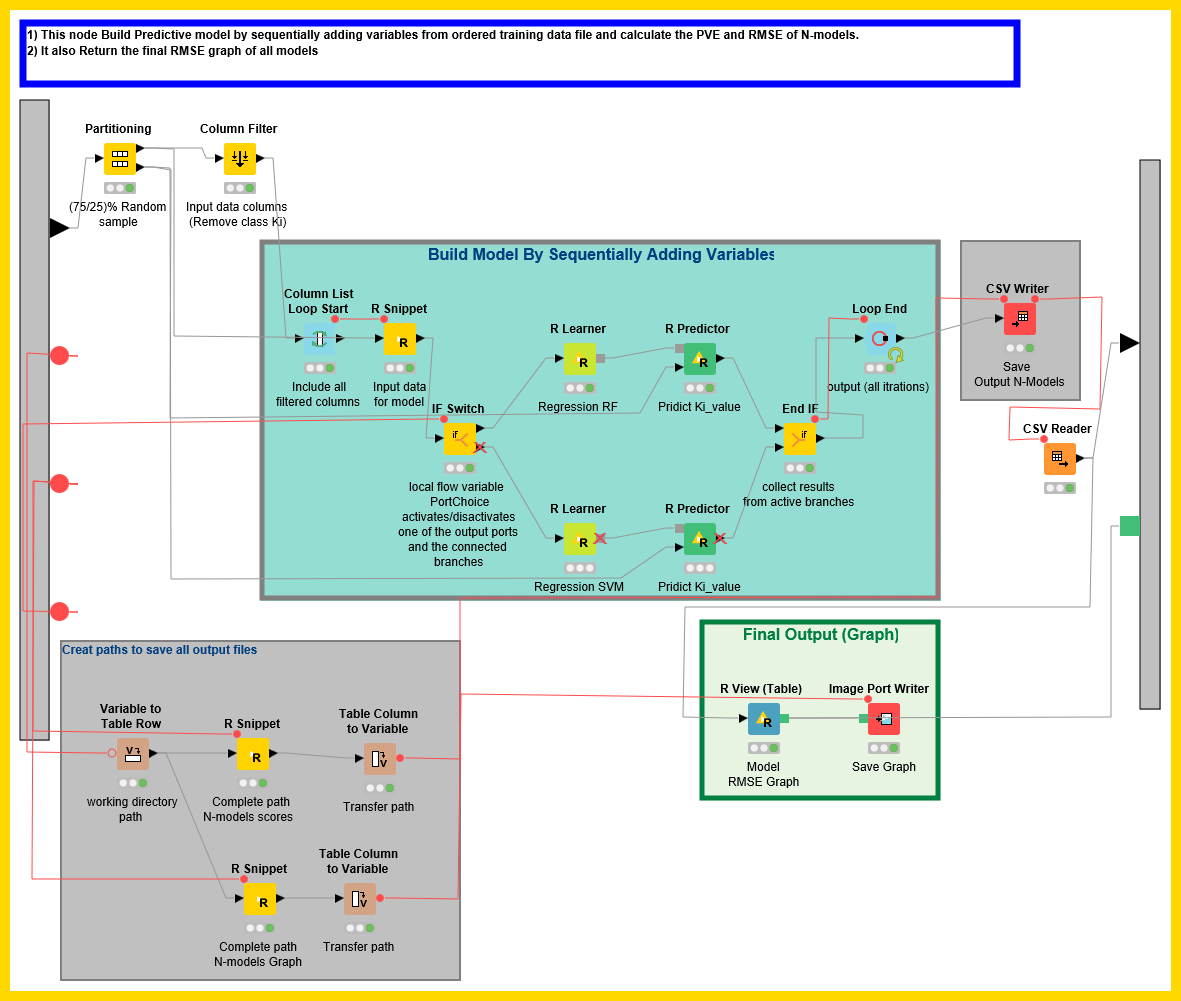

Supplement: Supplementary file 1 — Additional file 1. Figure S1. KNIME overview of automated QSAR modeling workflow. Figure S2. ChEMBLdb meta node. Figure S3. Nfold cross validation meta node. Figure S4. MeanMSE meta node. Figure S5. Stepwise estimation models meta node. Figure S6. Output files generated by automated QSAR modeling workflow. Figure S7. Mispredictions of over-fitted models. Table S1. Data modelability measure (MODI_ssR2) versus QSAR_PVE for 30 datasets. [file 13321_2017_256_MOESM1_ESM.zip › Additional file 1/Figure S5.png]

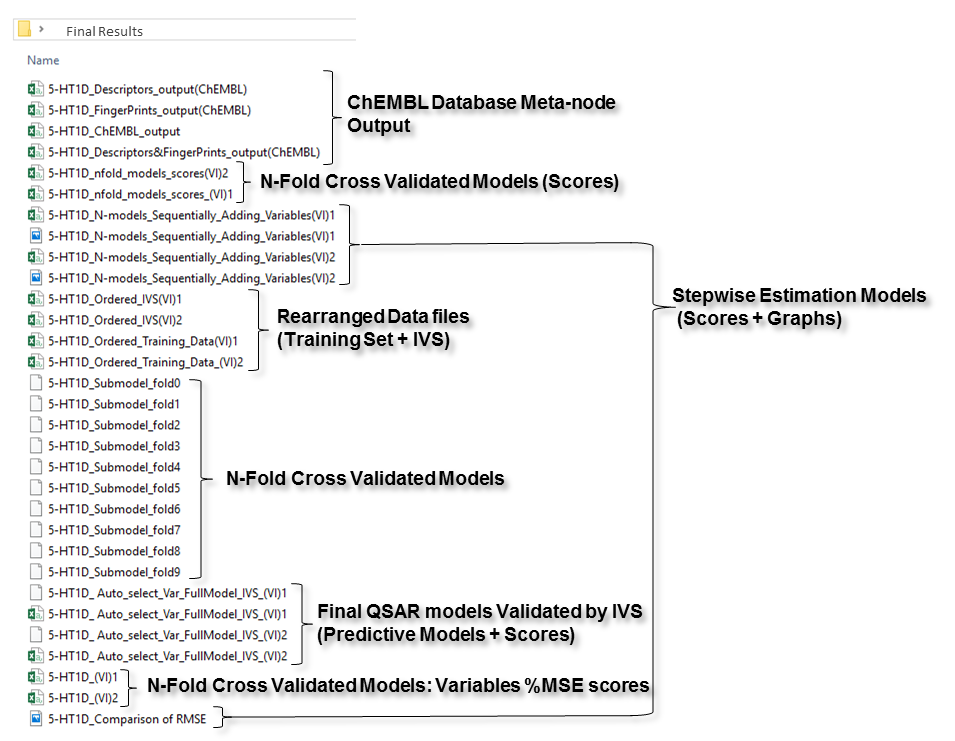

Supplement: Supplementary file 1 — Additional file 1. Figure S1. KNIME overview of automated QSAR modeling workflow. Figure S2. ChEMBLdb meta node. Figure S3. Nfold cross validation meta node. Figure S4. MeanMSE meta node. Figure S5. Stepwise estimation models meta node. Figure S6. Output files generated by automated QSAR modeling workflow. Figure S7. Mispredictions of over-fitted models. Table S1. Data modelability measure (MODI_ssR2) versus QSAR_PVE for 30 datasets. [file 13321_2017_256_MOESM1_ESM.zip › Additional file 1/Figure S6.png]

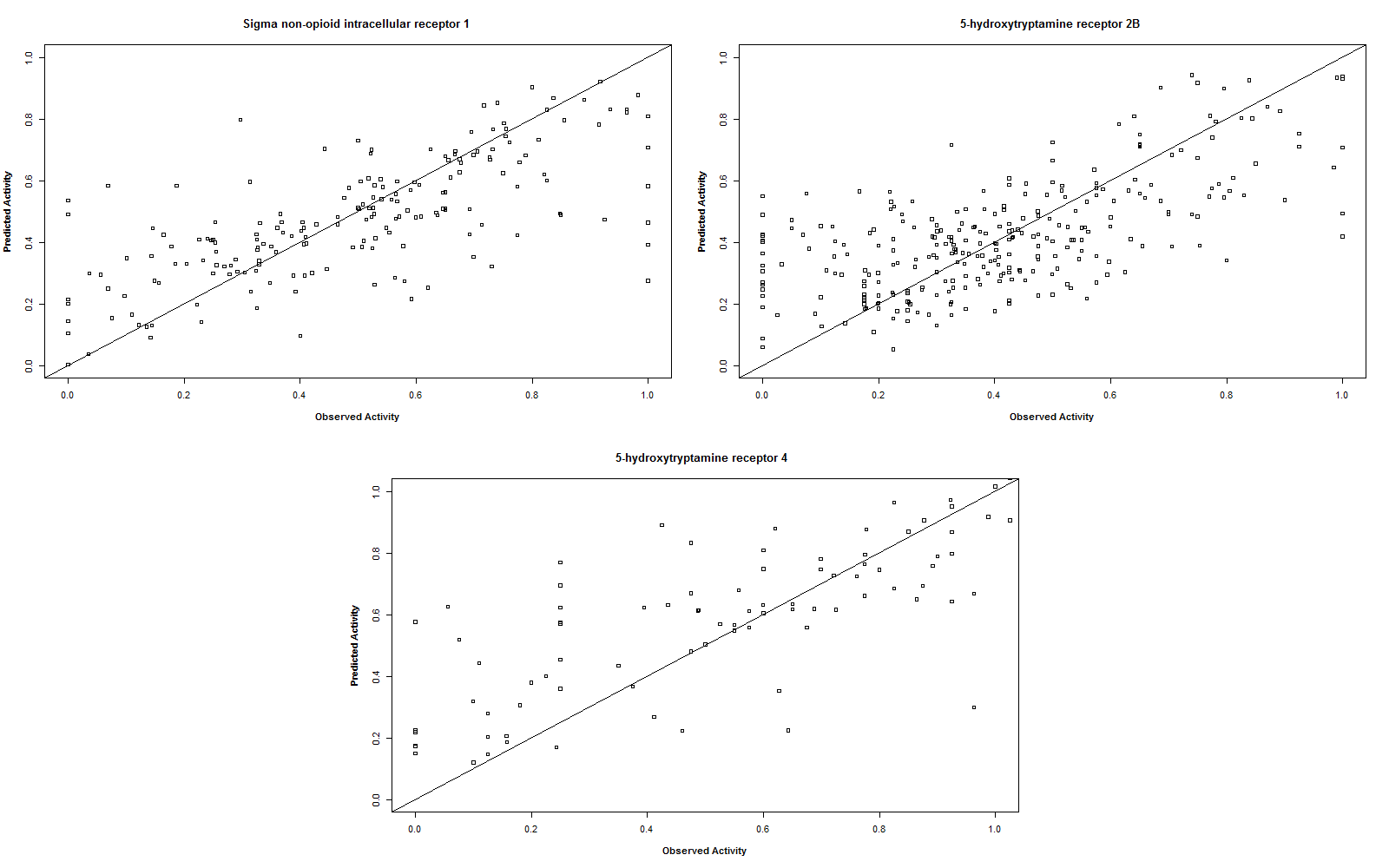

Supplement: Supplementary file 1 — Additional file 1. Figure S1. KNIME overview of automated QSAR modeling workflow. Figure S2. ChEMBLdb meta node. Figure S3. Nfold cross validation meta node. Figure S4. MeanMSE meta node. Figure S5. Stepwise estimation models meta node. Figure S6. Output files generated by automated QSAR modeling workflow. Figure S7. Mispredictions of over-fitted models. Table S1. Data modelability measure (MODI_ssR2) versus QSAR_PVE for 30 datasets. [file 13321_2017_256_MOESM1_ESM.zip › Additional file 1/Figure S7.png]
